# Bound or unbound: mapping and monitoring receptor oligomerization by time-resolved fluorescence live-cell imaging

**DOI:** 10.3389/fmolb.2026.1814859

**Published:** 2026-06-16

**Authors:** Annemarie Greife, Ruiqi Liu, Paul S. Köhler, Katrin G. Heinze, Katherina Hemmen, Thomas-Otavio Peulen

**Affiliations:** 1 Molecular Physical Chemistry, Heinrich Heine University Düsseldorf, Düsseldorf, Germany; 2 South China Biodiversity Research Center, School of Life Sciences, Guangzhou University, Guangzhou, China; 3 Rudolf-Virchow-Center for Integrative and Translational Bioimaging, Julius-Maximilians-University Würzburg (JMU), Würzburg, Germany

**Keywords:** FLIM (fluorescence lifetime imaging), GPCR, MC4R, live-cell FRET spectroscopy, homoFRET, protein-protein interactions, membrane protein affinities

## Abstract

Understanding protein oligomerization in living cells is essential for elucidating cellular signaling and regulation, yet quantitative analysis remains challenging due to heterogeneous expression levels, dynamic interactions, and limited access to absolute protein concentrations. Here, we present a standardized, open-source framework for quantifying protein assemblies in living cells by integrating Förster Resonance Energy transfer (FRET), fluorescence lifetime and anisotropy imaging (heteroFRET and homoFRET) with molecular brightness-based concentration estimation and image analysis. Using natural variants of a vertebrate GPCR, the melanocortin-4 receptor (MC4R-A and MC4R-B2), as a model system, we demonstrate how to discriminate monomers, dimers, and higher-order oligomers, extract inter-fluorophore distance distributions, and determine association constants under physiologically relevant conditions in living cells. Standard fluorescent protein tags report on proximity and oligomerization via Homo- and HeteroFRET. Association constants are quantified using the variable protein expression in living cells and the spectroscopy readouts. By high-content imaging we overcome the biological noise and attain data qualities comparable to conventional biochemical *in vitro* assays. Intensity- and fluctuation-based segmentation further extends the accessible concentration range within individual cells, improving affinity analysis robustness. Our results establish quantitative image spectroscopy on living cells as quantitative tool for investigating protein-protein interactions under physiologically relevant conditions. All computational workflows are implemented in open-source software and are accompanied by detailed protocols and analysis scripts, enabling reproducible application and adaptation. Beyond GPCRs, this framework provides a practical and transferable methodology for quantitative studies on protein-protein interactions, mechanistic studies and drug discovery in complex cellular environments.

## 1 Introduction

Proteins orchestrate nearly every biological function. In many signaling processes in and between cells, individual proteins assemble into protein complexes. Thus, it is essential to understand the composition and interaction dynamics of such protein complexes to better disclose their biological and physiological roles (Marsh and Teichmann, 2015). Classical approaches such as yeast two-hybrid (Duarte and Euclydes, 2024), co-immunoprecipitation (Tan and Yammani, 2022), and pull-down assays (Gnanasekaran and Pappu, 2023) provide highly valuable information on protein-protein interactions (PPI). These classic approaches are efficient and successful for soluble cytoplasmic or nuclear proteins, but can be challenging for transient interactions, and membrane-bound proteins (Zhang et al., 2025). Here, Förster Resonance Energy Transfer (FRET) (Förster, 1948) offers a clear advantage, as interactions in living cells can be spatially monitored in real-time (Bonilla and Shrestha, 2024; Sun et al., 2012; Zhang, et al., 2025).

Among all membrane-bound proteins, the largest family are the G protein-coupled receptors (GPCR), which are important pharmacological drug targets. About 35% of approved drugs so far target GPCRs or GPCR-related proteins (Calebiro and Grimes, 2020; Sriram and Insel, 2018). Melanocortin 4 receptor (MC4R) is a class A GPCR involved in metabolism and obesity in humans (Farooqi et al., 2003). MC4R mainly expresses in the central nervous system, particularly in the hypothalamus (Wei et al., 2023). It plays a key role in energy homeostasis, including both food intake and energy expenditure. The MC4R system is highly conserved across vertebrates, from fish to mammals (Tao, 2022). In *Xiphophorus* fish, commonly known as platy fish and swordtails, the highly polymorphic and sex-linked *mc4r* locus is the key genetic factor controlling the timing of puberty and, as a result, the ultimate body size in males (Lampert et al., 2010; Volff et al., 2013). This leads to a remarkable divergence in expression within their natural population. In two swordtails, *Xiphophorus nigrensis* and *Xiphophorus multilineatus*, onset of puberty is attributed to the ratio of the *mc4r* alleles A, B1, and B2, and their copy number variation on the X- and Y-chromosomes (Lampert, et al., 2010). The wild-type A allele has full signaling capacity, while the mutant B alleles, B1 and B2, cannot be stimulated by agonists. Unlike A allele, B1 and B2 lack the C-terminal dicysteine motif and are unable to anchor the C-terminal helix VIII into the plasma membrane. B2 has another four-base deletion, resulting in an elongated C-terminus (Lampert, et al., 2010).

It has been recognized that class C GPCRs are fully functioning only as dimers to transduce signals (Kniazeff et al., 2011); yet for class A GPCRs, such as MC4R, debate remains if and to which extent they form dimers or oligomers, and what function the dimer/oligomer confers (Milligan et al., 2019). *In vitro* analyses only provide limited information about interaction in living cells (Zhang, et al., 2025). To study PPIs in living cells, we combine fluorescence spectroscopy with high-content imaging and establish open-source analysis workflows for quantitative PPI analysis (Peulen, 2025; Peulen et al., 2025), and provide step-by-step protocols (Greife et al., 2025). As benchmarks to investigate oligomerization states, we use two *Xiphophorus* MC4R variants, A and B2, tagged with standard fluorescent proteins (FPs) at the C-terminus, representing receptors with respective short and long peptide chains linking to the FPs (Lampert, et al., 2010; Liu et al., 2023). With this receptor model, we apply time-resolved FRET to measure GPCR proximity via Homo- and Hetero-FRET.

Here, we use FRET as a measure for the (sub-)nanometer distance between MC4R molecules. FRET informs on distances between donor and acceptor fluorophores (here: fluorescent protein-tagged MC4R). Generally, FRET is used as a specific indicator for molecular interactions. FRET, a non-radiative energy transfer from a donor to an acceptor fluorophore, can report on inter-fluorophore distances (Förster, 1948). The efficiency of that transfer process, *E*, depends on the sixth power of the inter-fluorophore distance (*R*_*DA*_). Thus, FRET happens typically at distances below 10 nm. Albeit, at high concentrations, FRET may occur even in the absence of a direct interaction due to molecular proximity (Wolber and Hudson, 1979). Nevertheless, as a molecular ruler, FRET is uniquely suited to report molecular proximity and conformational or interaction changes in living cells (Sun, et al., 2012; Weidtkamp-Peters et al., 2009; Zhang, et al., 2025).

Classical intensity-based FRET approaches often suffer from spectral bleed-through, cross-excitation, and variable fluorophore expression levels (Bonilla and Shrestha, 2024). Time-resolved, fluorescence lifetime-based FRET implemented through time-correlated single photon counting (TCSPC) or frequency-domain fluorescence lifetime imaging (FLIM) mitigate these limitations by directly measuring FRET-induced donor lifetime shortening (Bonilla and Shrestha, 2024; Weidtkamp-Peters, et al., 2009). FLIM-based FRET is largely independent of fluorophore concentration, photobleaching, or excitation intensity and therefore provides a quantitative, calibrated approach to probe interaction dynamics in heterogeneous samples such as the plasma membrane (Greife et al., 2016), cellular compartments (Kravets et al., 2016), or in crowded cellular microdomains (Lou et al., 2024).

Depending on fluorophore configuration, two types of FRET experiments inform on PPIs. In heteroFRET experiments, distinct donor and acceptor fluorophores inform on pairwise interactions. They are particularly useful for mapping binary associations and the fraction of interacting molecules (Greife, et al., 2016; Kravets, et al., 2016; Weidtkamp-Peters, et al., 2009; Zhang, et al., 2025). In homoFRET experiments, the energy transfer between identical fluorophores is monitored. The energy migration between two identical fluorophores manifests as a decrease of fluorescence anisotropy. HomoFRET experiments allows single-color detection of oligomer states (Chan et al., 2011; Warren et al., 2015) and are therefore suited to quantify concentration-dependent oligomerization or to distinguish monomers from dimers/oligomers (Chan, et al., 2011). HomoFRET experiments are technically and analysis-wise more demanding, because the time-resolved fluorescence anisotropy decays are influenced by rotational diffusion, local viscosity, and photoselection; consequently, robust decay models including the required corrections and appropriate instrument corrections are essential to extract reliable donor-donor energy transfer rates (Vogel et al., 2015; Vogel et al., 2009).

Until recently, the implementation of quantitative FRET/FLIM - especially time-resolved anisotropy for homoFRET - required proprietary software or scripting, and substantial expert knowledge. This limited a broad adoption, particularly when access to specialized spectroscopy microscopes and workflows is also limited. Over the last years, substantial progress has been made in open-source software ecosystems that integrate photon-counting, lifetime fitting, anisotropy decay modeling, ROI-based quantification, and spatially resolved FRET efficiency mapping. Packages such as FLIMfit (Warren et al., 2013), PAM (Schrimpf et al., 2018), ChiSurf (Peulen, 2025), and modular Python-based workflows, e.g., in *tttrlib* (Peulen, et al., 2025), now allow non-experts to perform data analysis and complex model fitting, correct for scattered light and objective depolarization, use model-free estimators such as *ε*(t), and incorporate segmentation or concentration maps directly into pixel-wise analysis. These developments enable reproducible, transparent oligomerization studies in live cells, lower the entry barrier for quantitative FRET/FLIM, and provide a foundation for standardized protocols. In this work, we build upon these recent advances and provide practical guidelines, quality checkpoints, and experimental considerations for reliable FRET-based oligomerization analysis using MC4R receptor variants as GPCR model system.

Previous studies revealed MC4R homodimerization and heterodimerization by FRET analysis, with MC4R-B2 exerting a dominant-negative effect on MC4R-A signaling (Liu et al., 2020; Liu, et al., 2023). Key controls using a monomeric reference (β1AR) and an obligate dimer (CD28) confirmed that the observed FRET signals exceed what is expected from random proximity alone. Thus, the MC4R-A and MC4R-B2 serve here as model receptors to develop and validate an analysis workflow that prevents misinterpretation in FRET binding assays. Conceptually, this work establishes a practical and quantitative framework for analyzing protein oligomerization in living cells by integrating time-resolved heteroFRET and homoFRET, molecular brightness-based concentration estimation, and image segmentation. By combining these complementary modalities within an open-source analysis workflow, we overcome technical hurdles in high-content image spectroscopy and key limitations of conventional FRET approaches, including uncertainty in protein concentration, heterogeneous expression, and ambiguity in oligomeric state assignment. Our approach enables the direct extraction of apparent association constants and inter-fluorophore distance distributions under physiologically relevant conditions, while maintaining experimental and computational accessibility. Beyond the specific case of GPCRs, this framework provides a methodology for quantitative studies of protein-protein interactions in cells. Critically, this integration enables quantitative insights, including apparent association constants, inter-fluorophore distance distributions, and oligomeric state discrimination, that are not accessible through conventional single-readout FLIM workflow. In short, this integration enables the extraction of oligomerization equilibria directly in living cells.

## 2 Materials and methods

### 2.1 Sample preparation and data acquisition

#### 2.1.1 Plasmid preparation

The genes for *mc4r-A* and *B2* have been cloned previously into a pcDNA3.1 backbone between *HindIII* and *XbaI* while eGFP and mCherry, respectively, were cloned C-terminally using *XbaI* and *NotI* restriction sites (Lampert, et al., 2010; Liu, et al., 2023). No further amino acids were inserted as linkers between MC4R and the fluorescent protein (Supplementary Table S1).

#### 2.1.2 Cell culture

Human embryonic kidney 293T (HEK293T) cells were cultured in Dulbecco’s modified Eagle medium (DMEM) without sodium pyruvate (P04-03550, PAN Biotech, Aidenbach, Germany), with 10% fetal calf serum (AC-SM-0190, Anprotec, Bruckberg, Germany) and 100 U/mL penicillin, 100 μg/mL streptomycin (P4333, Sigma-Aldrich, St. Louis, Missouri, United States) at 37 °C, 5% CO_2_. To passage the cells, culture medium was aspirated; cells were washed with PBS (14190, Gibco, Thermo Fisher Scientific, Waltham, Massachusetts, United States) once, and cells were detached using 0.5× Trypsin-EDTA solution (T4299, Sigma-Aldrich). The cell suspensions were resuspended in DMEM culture medium. Cells were regularly passaged every 3–4 days.

#### 2.1.3 Live-cell fluorescence lifetime imaging (FLIM)

FLIM was performed on live-cell samples. For accurate measurements, the fluorescent background in the cells must be minimal (e.g., usage of Phenol-red free media). Thus, we recommend assessing growth and imaging conditions by imaging non-transfected cells. Under our imaging conditions, fluorescence count rates within the transfected cells were several orders of magnitude higher than in non-transfected cells*.* HEK293T cells were seeded at 75,000 cells/well on poly-D-lysine coated 4-chambered coverglasses (C4-1.5P, Cellvis, Mountain View, California, United States) and grown for ∼48 h. Cells were transiently transfected by jetPRIME using 1 µL jetPRIME reagent and 50 µL jetPRIME buffer (Sartorius Sartorius GmbH and Co. KG, Goettingen, Germany) according to the manufacturer’s instructions with either an eGFP-tagged construct for DO samples or a mixture of eGFP- and mCherry-tagged constructs for the FRET samples. In each sample, a total of 0.5 µg DNA was used in a transfection volume of 500 µL DMEM. Empty backbone vector was mixed with MC4R constructs to keep the total DNA amount constant. For DO samples, 0.25 µg eGFP-tagged constructs were mixed with 0.25 µg empty pcDNA3 vector. For FRET samples, the ratio between eGFP- and mCherry-tagged construct varied from 1:1 to 1:20. The cell culture medium was changed at six to 8 hours post-transfection, and the cells were incubated overnight. On the next day, medium was changed to imaging medium [DMEM without phenol red (P04-01161, PAN Biotech) supplemented with 15 mM HEPES (15630-080, Gibco), 10% fetal calf serum, and 2 mM glutamine (G7513, Sigma-Aldrich)], which allows for 2–3 h of live-cell imaging without a CO_2_ source.

##### 2.1.3.1 Setup

FLIM was performed using a Zeiss LSM980 confocal microscope (Zeiss, Oberkochen, Germany), operated with ZEN3.3 software and equipped with the LSM upgrade kit (PicoQuant, Berlin, Germany), which is managed by SymPhoTime64 software. Both systems are controlled through the Zeiss Blue PicoQuant Application software. The optical setup must be optimized for the sample. For experiments in living cells, we used a high-NA water objective (40×/1.2 NA w). Experiments on fixed cells embedded in the fixation medium can be performed with high-NA oil objectives. However, oil objectives lead to more depolarization (Supplementary Figure S12) and are less suitable for reliable homoFRET experiments. The axial contribution of intracellular fluorescence was assessed by volumetric imaging (Supplementary Figure S1). The excitation was achieved using pulsed lasers at 485 nm and 560 nm (LDH-P-C-485B/LDH-D-TA-560B, PicoQuant, Berlin, Germany). The emitted fluorescence photons were first split by a polarizing beam splitter, followed by a 560 nm long pass (560/LPXR, AHF, Tübingen, Germany) to separate green and red signals. Green photons passing HC520/35 bandpass filters (Semrock, New York, U.S.A.) were registered by PMA Hybrid 40 detectors (PicoQuant, Berlin, Germany). Red photons passing an ET600/50 bandpass filter (Chroma Technology GmbH, Olching, Germany) were detected by Excelitas SPADs (SPCM-AQRH-14-TR, PicoQuant, Berlin, Germany).

##### 2.1.3.2 Image acquisition

Images were acquired at a zoom factor of 6.0 resulting in a pixel size of 0.069 µm. The image dimensions were set at 512 × 512 pixels, and the data was collected at a pixel dwell time of 8.16 µs over the course of 50 frames.

##### 2.1.3.3 FLIM measurement

During the measurement, the repetition rate was set to 40 MHz. Pulsed interleaved excitation (PIE) (Muller et al., 2005) was utilized at a delay time of 25 ns and for a time window of 50 ns to modulate donor and acceptor excitation. The excitation laser power was adjusted between 200 and 800 nW at 480 nm and 140 and 550 nW at 560 nm. The time-correlated single photon counting (TCSPC) resolution was 10 ps.

##### 2.1.3.4 Calibration procedure

Before live-cell measurements, the setup was calibrated following a standard routine. Initially, the instrument response function (IRF) was determined using Erythrosine B (200964, Sigma-Aldrich) in a saturated potassium iodide (KI) solution (221945, Sigma-Aldrich). Daily fluorescence correlation spectroscopy (FCS) and TCSPC data acquired for the dyes Alexa488 and Alexa568 and the fluorescent proteins eGFP and mCherry served as molecular brightness and diffusion standards to determine molecular concentrations, the instrumental g-factor and polarization mixing factors (Erdelyi et al., 2014; Koshioka et al., 1995).

#### 2.1.4 Volumetric imaging of fixed cells

For comparative volumetric imaging of MC4R transfected HEK293T cells, the cells were transfected as described above. After the overnight incubation, the cells were washed twice with PBS prior to fixation with 4% paraformaldehyde (J61899, ThermoFisher Scientific) for 15 min at room temperature. The cells were washed twice PBS followed by 5 min nuclear staining with a 1:3000 dilution of HOECHST 33342 (stock: 10 mg/mL, B2261, Merck KGaA). The cells were washed thrice with PBS and directly imaged on the Zeiss LSM 980. The fluorescence signal was collected in two subsequent tracks (Track 1: eGFP [excitation 488 nm, emission 499–578 nm, GaAsP-PMT], Track 2: HOECHST [excitation 405 nm, emission 408–495 nm, PMT] and mCherry [excitation 561 nm, emission 578–693 nm, GaAsp-PMT]). The excitation intensities were adjusted per sample. The 3D stacks were acquired with a 53 × 53 × 160 nm step size and a scan rate of 2.8 µs/pixel using bidirectional scanning.

#### 2.1.5 Simulation of fluorescent protein distributions

The sterically allowed distribution of fluorescent proteins, FPs, coupled to MC4R dimers were simulated using the integrative modelling platform, IMP (Russel et al., 2012). Given the amino acid sequences, dimeric structures of MC4R with attached FPs were created with AlphaFold3 (Abramson et al., 2024). To effectively sample the sterically allowed conformational space, the structures were coarse-grained in IMP on residue-level. Residues and groups of residues are represented by beads. In the coarse-grained representation MC4R dimers and FPs were represented by rigid bodies, whereas the linker coupling the FPs to the MC4R dimer was represented by a string of flexible beads. In rigid bodies, relative distances across beads are constrained during conformational sampling. In flexible strings of beads, the beads are restrained by the sequence connectivity (Algret et al., 2014). In the sampling, excluded volume restraints (Shi et al., 2014) were applied in addition to a knowledge-based membrane restraint localizing the MC4R dimer into a membrane and excluded the FPs and the linker from the membrane. The sterically allowed models were sampled by Gibbs sampling, based on the Metropolis Monte Carlo algorithm. FP, MC4R, and bead positions were randomized followed by a brief gradient descent optimization. Next, Monte Carlo moves included random translation and rotation of rigid bodies (up to 1 Å and 0.01 radians, respectively), and random translation of individual beads in the flexible segments (up to 2 Å) were used for sampling. For each computed model, the inter FP distance, $R_{DA}$ , and the orientation factor, $\kappa^{2}$ , were calculated.

### 2.2 Data analysis

A complete step-by-step data analysis, including all required analysis scripts and example data, has been deposited on zenodo (https://doi.org/10.5281/zenodo.17869433). The complete dataset containing the raw data and the segmentation masks have been deposited as well (https://doi.org/10.5281/zenodo.18175060). Based on this data and the provided script the complete analysis can be reconstructed.

#### 2.2.1 Live-cell fluorescence lifetime imaging (FLIM)

Our time-resolved Förster Resonance Energy Transfer (FRET) analysis workflow consists of several steps (outlined in detail below). Briefly, the registered photons were binned into pixels to yield images. Registered photons encode information on detectors and their arrival time with picosecond resolution. We compute pixel-wise intensities (intensity images) and mean photon arrival times relative to the excitation pulse as average features that we map to images. These features we use to identify cells, segment images, and to define regions of interest (ROIs). Finally, we integrate photons of ROIs into fluorescence intensity decay histograms (fluorescence decay). Integrating photons of ROIs decreases the noise of fluorescence decays and enables fitting of the fluorescence lifetimes and inter-fluorophore distances. More details on the data analysis can be found in the Supplementary Information.

##### 2.2.1.1 Export of intensity images

Fluorescence intensity images were generated by integrating photons of all frames in the three channels: green-prompt (donor emission), red-prompt (FRET-sensitized acceptor emission), and red-delay (directly excited acceptor emission) and exported as 16-bit tiff-files using *tttrlib* (Peulen, et al., 2025) and *scikit-image* (van der Walt et al., 2014).

##### 2.2.1.2 Image segmentation

Cells and regions of interest (ROIs) were segmented from the summed intensity of all three channels using median filtering (two-pixel radius), followed by Li thresholding, both implemented in *scikit-image* (Li and Lee, 1993; Li and Tam, 1998). Small holes (200 pixels) were filled and small objects (<10,000 pixels) removed. These parameters were determined on representative datasets and kept constant across experiments. Segmentations were curated in Fiji (Schindelin et al., 2012). The effect of out-of-focus fluorescent signals can be estimated by volumetric imaging. We studied these effects by 3D confocal stacks acquired using the same microscope and objective (Zeiss LSM980, 40×/1.2 NA w). This allowed us to quantify the fluorescent signal and consider the optical sectioning of the microscope. Under our imaging conditions, the signal is dominated by the basal membrane compared to the intracellular signal (Supplementary Figure S1). Nevertheless, in our images, we identify fast-moving vesicle-like structures, classified in a separate pixel class in the sub-region segmentation. Photons from this class can be discarded from the analysis, depending upon the research question.

##### 2.2.1.3 Sub-region segmentation

ROIs were further divided into five sub-ROIs using two methods. First, vesicles identified in the red-delay channel were segmented using ilastik (Berg et al., 2019), excluded from the cell mask, and the remaining area split into “low” and “high” intensity regions by Otsu thresholding (Otsu, 1979). Second, the Number and Brightness (N&B) approach (Digman et al., 2008), was applied to intensity fluctuations across all channels to segment cells into “low B” (B < 1.375, predominantly monomeric) and “high B” (B ≥ 1.375, predominantly dimeric) regions. All sub-regions were exported as 8-bit binary tiff masks. The threshold of B = 1.375 is independent of the eGFP:mCherry expression ratio, as both monomeric eGFP and monomeric mCherry individually exhibit B ≈ 1; values exceeding this threshold reflect correlated intensity fluctuations arising from molecular complexes regardless of their fluorophore composition.

##### 2.2.1.4 Export of fluorescence intensity decays

Fluorescence decay histograms were computed for all channels (green-prompt, red-prompt, red-delay) from the segmented ROI masks using *tttrlib* and *scikit-image*, with a micro-time bin size of 20 ps (twofold binning). Parallel and perpendicular detection channels were combined into a single column.

##### 2.2.1.5 Determination of correction factors

Measurements used vertically polarized excitation with parallel (VV) and perpendicular (VH) detection. To correct for polarization mixing introduced by high-NA objectives and for detector sensitivity imbalances, the *g*-factor, *l*_*1*_, and *l*_*2*_ were determined by jointly analyzing fast-rotating dyes (Alexa488, Alexa568) and slowly rotating fluorescent proteins in *ChiSurf* (Erdelyi, et al., 2014; Koshioka, et al., 1995; Peulen, 2025). The green channel yielded *g* = 0.946, *l*_*1*_ = 0.059, *l*_*2*_ = 0.305; the red channel yielded *g* = 0.995, *l*_*1*_ = 0.121, *l*_*2*_ = 0.279.

##### 2.2.1.6 Determination of average fluorescence lifetimes

Average fluorescence lifetimes of eGFP in donor-only (DO) and FRET (DA) samples were determined in ChiSurf by iterative reconvolution of multi-exponential decay models to the measured fluorescence intensities (Supplementary Equations S1, S2), accounting for the IRF, polarization mixing, and detector sensitivities. Species-averaged lifetimes were computed according to Supplementary Equation S3.

##### 2.2.1.7 Determination of inter-fluorophore distances (heteroFRET)

Donor fluorescence decays were fitted with a mixture model containing non-FRET and FRET-active species (Supplementary Equation S4), with the FRET-induced donor decay related to a Gaussian inter-fluorophore distance distribution centered at mean distance *R_app_* with half-width *σ_app_* (Supplementary Equations S5, S6) (Peulen et al., 2017). FRET rate constants were computed assuming κ^2^ = 2/3 and a Förster radius of R_0_ = 52 Å for the eGFP/mCherry pair (Lambert, 2019), yielding apparent distances that reflect both spatial separation and orientational averaging. Analysis proceeded in three rounds: first, fitting entire-cell decays with a single Gaussian; second, fitting with a mixture of two Gaussians, where a short, narrow component (σ ≈ 1 Å) captures collective quenching by multiple acceptors in oligomeric assemblies (Greife, et al., 2016; Kravets, et al., 2016), and a longer distance component represents the MC4R dimer; third, fixing the recovered distance parameters to determine species fractions for entire cells and the five segmented sub-ROIs. For an overview of fit models see Supplementary Table S3. Exemplary fluorescence decay histograms of the different fit models are shown in Supplementary Figure S3.

##### 2.2.1.8 Inter-fluorophore distances determined by homoFRET

In homoFRET, energy transfer between identical fluorophores causes additional fluorescence depolarization, with the anisotropy relaxation time decreasing with fluorophore proximity and the fundamental anisotropy amplitude lowering accordingly. Parallel and perpendicular fluorescence decays were jointly fitted using bi-exponential (dimer) and tri-exponential (oligomer) anisotropy models (Supplementary Equations S7, S8), with species fractions for monomer, dimer, and oligomer and their corresponding FRET rate constants recovered as fit parameters. The anisotropy relaxation times are coupled to the global rotational correlation time of the FP-tagged receptor, estimated at ∼100 ns (compared to ∼12 ns for free FPs; (Striker et al., 1999)), and FRET-rate constants were converted to apparent inter-fluorophore distances via the Förster equation (Supplementary Equations S9–S11), using R_0_ = 49 Å and τ_0_ = 2.38 ns for eGFP, and R_0_ = 44 Å and τ_0_ = 1.31 ns for mCherry (Lambert, 2019). For simplicity, discrete relaxation times rather than distance distributions were fitted. For an overview of fit models see Supplementary Table S4. All corrections (g-factor, polarization mixing, background) are incorporated at the model level during fitting. Raw photon counts are not modified, preserving Poisson statistics.

Steady-state anisotropies were additionally computed for eGFP in donor-only and eGFP/mCherry samples, and for directly excited mCherry, from background-corrected parallel and perpendicular count rates (Supplementary Equation S12), with backgrounds estimated from fitted decay offsets.

#### 2.2.2 Concentration estimation: converting photon counts to concentration

To relate dimer and oligomer fractions to MC4R expression levels, fluorophore concentrations were determined via Fluorescence Correlation Spectroscopy (FCS) on singly transfected cells (MC4R-eGFP or MC4R-mCherry). Molecular brightness values used for concentration estimation were determined by FCS calibration measurements under conditions matched to FLIM acquisition. As these parameters are instrument- and setup-dependent, they were determined specifically for the experimental configuration used in this study. Concentrations were then derived from background-corrected average count rates in each ROI (Supplementary Equations S13–S17 (Hemmen et al., 2021)). Background contributions were estimated from decay offsets. Background-corrected count rates were used for concentration and brightness estimation. Constant background contributions reduce correlation amplitude and were accounted for accordingly. For eGFP in FRET samples, count rates were additionally corrected for FRET-induced quenching using the transfer efficiency E and FRET-active fraction x_FRET_ from the Gaussian distance fits (Supplementary Equations S5, S6), before converting to concentration (Supplementary Equations S16, S17). The donor-to-acceptor concentration ratio was defined as a key parameter governing the observed FRET amplitude (Supplementary Equation S18). Example FCS curves are shown in Supplementary Figure S4.

##### 2.2.2.1 Estimation of the association constants for oligomerization

MC4R dimerization and oligomerization were described by a stepwise equilibrium model in which dimers form first, followed by tetramerization as a formal representation of higher-order oligomers (Greife, et al., 2016; Kravets, et al., 2016). For any given total protein concentration, monomer, dimer, and oligomer species fractions were determined by solving the equilibrium equations to yield the apparent dimerization constant $K_{Dimer}^{*}$ and oligomerization constant $K_{Oligomer}^{*}$ . Since heteroFRET detects only mixed donor–acceptor complexes while donor–donor and acceptor–acceptor species remain invisible, the extracted association constants represent apparent *K*_*D*_ values based on a simplified binding isotherm that does not explicitly account for all complex species.

##### 2.2.2.2 Estimation of the dimerization constant from homoFRET analysis

An apparent dimerization constant was estimated by combining donor-only (DO) and directly excited acceptor (dir. A.) anisotropy results. Since acceptor decays were best described by a dimer model and DO samples by an oligomerization model, dimer and oligomer fractions from the DO analysis were merged into a single non-monomer fraction, and $K_{Dimer,pooled}^{*}$ was obtained by solving the resulting two-species equilibrium (Supplementary Equations S22, S23).

95% confidence intervals for the dimerization and oligomerization constants were determined using 500 bootstrap replicates.

#### 2.2.3 Pixel-based mean photon arrival times

Donor mean photon arrival times were computed pixel-wise from the micro-time distribution of stacked frames using *tttrlib* and *scikit-image*, retaining only pixels with more than 15 detected photons per frame. Results were exported as 32-bit float tiff images and masked with the binary ROI from the segmentation step for visualization.

#### 2.2.4 Statistical analysis

Statistical analyses were performed in OriginPro 2021b; (OriginLab Corporation, Northampton, MA, United States) using ANOVA. * = *p* < 0.05, ** = *p* < 0.01, *** = *p* < 0.001; individual cells were treated as independent observations, and no additional correction for experimental nesting was applied. Cells from at least three independent experiments were pooled.

#### 2.2.5 Excluded data

All data points underwent a 2-dimensional, multivariate location and scatter analysis. To detect outliers, the Mahalonobis distance was calculated (Etherington, 2021). Samples were excluded using a 97.5% threshold of the Mahalonobis distance determined from the monomer, dimer, and oligomer species fractions of the final global fit and the donor to total protein ratio. The analysis was performed separately for the MC4R-A and MC4R-B2 samples. We inspected the spatial distribution of excluded cells and found no systematic spatial pattern - excluded cells did not show preferential association with vesicle-rich regions or high mCherry intensity areas but mainly very low donor concentration. The exclusion was based solely on multivariate Mahalanobis distance thresholding of the species fractions and donor:total protein ratio, as described in the Methods. The excluded data points (9/119 for MC4R-A and 8/171 for MC4R-B2) were not carried into the segmentation-based analysis.

## 3 Results

### 3.1 FRET as a tool to identify protein-protein interactions

Wild-type MC4R receptors can form both homodimers, composed of two wild-type receptors, and heterodimers with mutant variants. This dimerization modulates intracellular signaling strength and may contribute to the variations observed in the timing of puberty (Lampert, et al., 2010; Liu, et al., 2020; Liu, et al., 2023). In this study, we build on the established knowledge of MC4R homodimerization (Kleinau et al., 2020; Liu, et al., 2023; Nickolls and Maki, 2006; Piechowski et al., 2013) to develop a detailed analysis workflow for live-cell experiments to (*i*) detect dimers and potential higher-order oligomers in live cells, (*ii*) estimate of association constants (*K*_*D*_), (*iii*) enhance discrimination between monomeric, dimeric, and oligomeric species using spectroscopic and image-derived features, and (*iv*) construct structural and geometric models.

**FIGURE 1:**
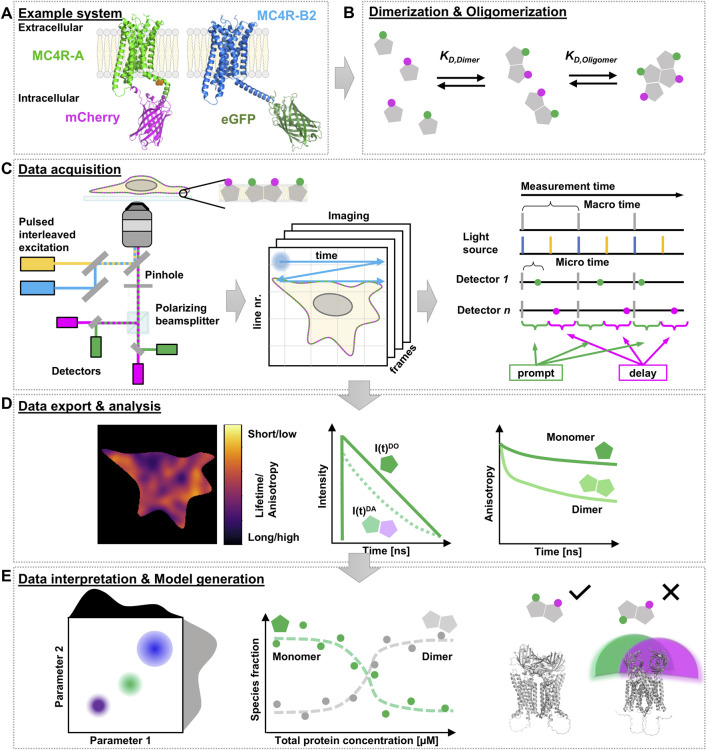
Fluorescence Lifetime Imaging (FLIM) combined with Pulsed Interleaved Excitation (PIE) resolves protein-protein interaction in the melanocortin-4 receptor (MC4R) by homo- or heteroFRET. **(A)** We focus on two out of three alleles from *Xiphophorus* MC4R, which differ in their intracellular C-terminal domain. In the A type (light green), two membrane anchors (orange) hold the intracellular C-terminal domain close to the membrane. In the B2 variant (blue), a frameshift causes the loss of the two membrane anchors, resulting in a much longer C-terminal tail. The MC4R structures were generated using AlphaFold3 (Abramson, et al., 2024). **(B)** C-terminally tagged constructs with either mCherry (magenta) or eGFP (green) (simplified as pentagons with magenta or green circles) are used for affinity and multimerization analysis: monomers are represented as pentagons, dimers and higher order oligomers are represented as connected pentagons. **(C)** In a confocal polarization-resolved PIE-FLIM setup (left), the donor (green, 485 nm laser) and the acceptor (magenta, 561 nm laser) excitation are alternated while the image is scanned (middle). The photons' arrival times relative to the experiment start (macro time) and the preceding laser pulse (micro time) are recorded, along with the detection channel (green/magenta, parallel/perpendicular) and image frame markers (right). **(D)** The information encoded in the photons is used to construct mean arrival times and steady-state anisotropy images (left). The integrated micro time of images or region of interest (ROIs) is analyzed to determine fluorescence lifetimes, inter-fluorophore distances (middle), or time-resolved anisotropies (right). **(E)** Joint analysis of multiple parameters and spatial information enables disentangling subcellular features (left). Exploiting variable expression levels allows for probing oligomerization and association constants (middle). Structural models of proteins (grey) and fluorophores (green/magenta) allow extraction of relative protein orientations and dimerization interfaces (right).

All constructs in this study are C-terminally fused to fluorescent proteins, either eGFP or mCherry (Figure 1A). Their unaltered activity and cellular localization were verified in cAMP assays (Liu, et al., 2023) and 3D confocal microscopy of fixed HEK293T cells (Supplementary Figure S1), respectively.

The wild-type MC4R-A possesses a comparatively short C-terminal tail that includes a post-translationally modified di-cysteine motif which anchors the cytoplasmic helix VIII to the cell membrane (Lampert, et al., 2010; Liu, et al., 2023). In contrast, the B2 variant contains a frameshift mutation that removes this motif and results in an extended C-terminal tail. Consequently, MC4R-A and -B2 differ in their flexible amino acid sequence (linker length), possibly altering the orientational (*κ* ^2^) and spatial freedom (accessible volume) of the attached fluorophores at the membrane.

Our analysis focuses on the homodimerization and potential higher-order oligomer formation of MC4R-A and MC4R-B2 (Figure 1B). To characterize these interactions, we performed fluorescence lifetime imaging (FLIM) in pulsed interleaved excitation (PIE) mode for both receptor variants (Muller, et al., 2005; Weidtkamp-Peters, et al., 2009). In live-cell PIE-FLIM, eGFP (donor) and mCherry (acceptor) are excited alternately on the nanosecond timescale: the donor is excited in the prompt time window, followed by direct acceptor excitation in the delayed time window (Figure 1C). Emitted fluorescence is collected through the same objective and separated first by polarization (vertical, VV, or horizontal, VH, relative to the excitation direction) and then by the emission color (donor vs. acceptor).

During data acquisition, the laser scans the image across multiple frames. For each detected photon, several parameters are recorded: (*i*) the detector identity (green/red and polarization channel), (*ii*) the macro time (time since acquisition started), and (*iii*) the micro time (time since the last prompt laser pulse). The macro time, together with encoded line and frame markers, allows assignment of photons to individual pixels. The micro time distinguishes whether a red photon originates from FRET-sensitized emission - indicating donor excitation followed by energy transfer - or from direct acceptor excitation in the delayed window.

Using these photon-resolved datasets, fluorescence lifetime and anisotropy images can be constructed (Figure 1D, left), and the time-resolved fluorescence intensity (Figure 1D, middle) and anisotropy (Figure 1D, right) can be obtained by summation of the respective photons. The shapes of these time-resolved curves already provide qualitative indications of energy transfer and thus protein interactions.

Quantitative analysis of pixel-wise fluorescence lifetimes, anisotropies, and count rates identifies cellular heterogeneities (Figure 1E, left) and supports the development of biophysical models, including the determination of dimerization association constants (Figure 1E, middle) and the inference of potential interaction sites within MC4R complexes (Figure 1E, right).

### 3.2 Identifying GPCR oligomerization

To identify the self-association potential of our two proteins of interest, we systematically varied the donor to acceptor ratios in titration experiments and performed live-cell FLIM measurements at the basal membrane of HEK293T cells. During transfection, we increased the number of mCherry-tagged receptors while maintaining a constant plasmid DNA quantity. Given that dimer and oligomer formation occur stochastically, this strategy increases the likelihood of forming FRET-active eGFP–mCherry receptor complexes.

**FIGURE 2:**
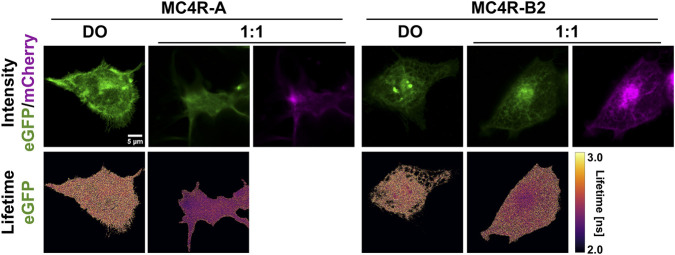
HEK293T cells co-transfected with MC4R eGFP and mCherry-tagged constructs show a decreased average fluorescence lifetime compared to singly transfected cells. (top) eGFP fluorescence intensity in the “prompt” time window of a singly transfected (“DO”) and mCherry fluorescence intensity in the “delay” time window (i.e., direct acceptor excitation) of a co-transfected cell with a 1:1 ratio of eGFP- and mCherry-tagged receptor (MC4R-A, left; MC4R-B2, right). (bottom) eGFP fluorescence lifetime images. The total amount of plasmid was kept constant. The average lifetimes were computed for pixels with at least 15 photons. Representative cells for all other transfection ratios are shown in Supplementary Figure S2. Scale bar = 5 µm.

**FIGURE 3:**
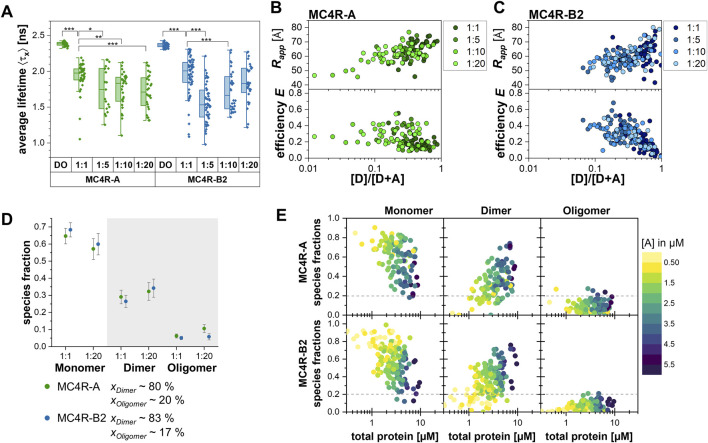
MC4R-A and MC4R-B2 variants show protein concentration-dependent oligomerization. A titration series of eGFP **(D)** and mCherry **(A)** tagged MC4R-A (green) and MC4R-B2 (blue) was performed with varying donor-to-acceptor (D:A) ratios. **(A)** The species-weighted average fluorescence lifetime ⟨*τ*⟩_x_ of D at different D:A ratios dropped from ∼2.4 ns to ∼1.7 ns as more acceptor, [A], was added. **(B,C)** The average apparent inter-fluorophore distance *R*_*app*_ (obtained from the single Gaussian distance fit), and FRET efficiency, *E*, varied with stoichiometry, [D]/[D + A]. **(D)** The mean and 95% confidence interval of the species fractions were obtained from fitting two Gaussian distances for both MC4R variants with DA 1:1 and DA 1:20 data (MC4R-A n = 56, MC4R-B2 n = 85). **(E)** Global analyses with two Gaussian distances reveal that the species fractions of both MC4R variants depend on the total protein and especially the acceptor concentration, [A] (MC4R-A: n = 116; MC4R-B2: n = 163; at least three independent experiments). The dashed lines at 20% species fractions indicate the threshold for oligomers. MC4R-mCherry concentrations are color-coded from 0.5 µM (yellow) to 6 µM (dark blue).

In the first analysis step, the fluorescence lifetime images generated by the microscope or exported via open-source software (Supplementary Figure S2) can be inspected visually. In this study, we used the open-source software *ttttrlib* (Peulen, et al., 2025) and *ChiSurf* (Peulen, 2025) for quantitative analysis. All scripts, data, and a step-by-step protocol used to produce the presented results are provided as supplementary material (Greife, et al., 2025). While large lifetime changes are readily detected in fluorescence lifetime images (Figure 2; Supplementary Figure S2), subtle differences or quantitative interpretation require more advanced analysis. Therefore, we defined regions of interest (ROIs) for each cell in each image. We exported the photon arrival times of the ROIs from the green channels, reconstructed time-resolved fluorescence intensity decays, and fitted them with a multi-exponential decay model to extract average fluorescence lifetimes (Supplementary Equation S1; Figure 3A). Note that our analysis relies on the image segmentation and grouping of photons with a typical fluorescence decay histogram of a segmented image containing (0.5–10 × 10^6^) photons. Contrary to traditional pixel-wise FLIM analysis, the data-noise is then negligible compared to the sample heterogeneity. For both MC4R-A and MC4R-B2, the average eGFP lifetime decreased from approximately 2.4 ns–1.7 ns as the fraction of mCherry-tagged receptor increased, indicating FRET and thus receptor-receptor interaction, consistent with specific binding rather than density-dependent proximity FRET, which we excluded in our previous study using monomeric (β1AR) and obligate dimeric (CD28) controls (Liu, et al., 2023).

Once protein-protein interactions have been confirmed, the next steps depend on the desired level of quantitative detail. The acquired data is higher dimensional. Affinity constants, *K*_*D*_, can be obtained in multiple ways. For instance, the observed fluorescence lifetimes can be plotted against either the receptor concentration (if available) or a suitable approximation, such as total count rate or the fraction of photons in the prompt window relative to all prompt and delayed photons. This ratio, known as stoichiometry, serves as a proxy for the donor/acceptor composition. Since expression levels and their ratio vary from cell to cell, transfection ratios are not reliable stoichiometry proxies; instead, donor-to-total protein ratios are calculated directly from per-cell fluorescence intensities. In a concentration-dependent dimerization model, the observed FRET efficiency depends on both the total concentration and the acceptor-to-donor ratio. As a result, the slope of FRET efficiency plotted against either parameter already provides initial insights into relative *K*_*D*_ values for the two MC4R variants (Figures 3B,C, bottom).

### 3.3 Quantifying GPCR oligomerization

Accurate and precise quantification of the oligomerization depends on the models’ and reference samples’ accuracy, and the data noise. It is reasonable to assume that the FPs coupled to MC4R adopt multiple conformations. Hence, to accurately interpret our experiments, we analyzed the fluorescence intensity decays using an inter-fluorophore distance model (Supplementary Equations S4–S6). The model describes interacting eGFP-mCherry pairs by a Gaussian distribution of width *σ*_*app*_ limited to a physical reasonable range (5-20 Å) and an average distance *R*_*app*_. The model includes a fraction $x_{noFRET}$ representing molecules that do not exhibit detectable FRET, comprising both truly monomeric receptors and associated receptors with unfavorable dipole orientations. Molecules not undergoing FRET are either (*i*) non-interacting, monomeric molecules (*x*_DO_) or (*ii*) fluorophore pairs with unfavorably oriented dipoles (*x*_Dipl_). Experimentally, we found that the donor fluorescence lifetime signatures (average lifetime >2.30 ns) of cells transfected for FRET with very low acceptor expression match the signature of the reference (eGFP-only constructs) cells (average lifetime 2.38 ns (MC4R-A) and 2.36 ns (MC4R-B2), Figure 3A) supporting the accuracy of our reference for analysis. To increase the precision and minimize the data noise, we stabilized our analysis by joint fits across multiple samples. The distribution width *σ*_*app*_ depends on the linker length, linker flexibility, and the local environment (e.g., membrane context).

The extracted distances and the FRET-inactive fractions were plotted against the concentration ratio (Figures 3B,C; Supplementary Figure S5A). For concentration-dependent trend analysis, we approximated $x_{noFRET}$ as the monomeric fraction, while acknowledging that this represents an upper bound due to orientation-induced FRET silencing. Thus, we neglected *x*_Dipl_ and treated *x*_noFRET_ as fraction of monomeric MC4R, *x*_*mo*_ (For more details on orientation effects, please refer to Section 3.8). When only dimerization occurs, the inter-fluorophore distance remains constant across the concentration range, while *x*_*mo*_ decreases (Supplementary Figure S5A). When higher-order oligomers form, the inter-fluorophore distances decrease while *x*_*FRET*_ increases concentration-dependent (Greife, et al., 2016). For MC4R-A, the average inter-fluorophore distance decreased from approximately 70 Å to 55 Å. For MC4R-B2 it decreases from approximately 75 Å to 50 Å (Figures 3B,C). These changes are a clear hallmark for the formation of higher-order oligomers.

These results prompted us to analyze the data with a three-state model comprising monomers, dimers, and oligomers. The dimer contribution was modeled with a Gaussian distance distribution. In the oligomeric state, varying receptor compositions with multiple donor-to-acceptor stoichiometries form. An eGFP-tagged receptor may be surrounded by one or more mCherry-tagged receptors, with each acceptor contributing independently to donor quenching. The collective FRET from multiple acceptors produces a notably high apparent FRET-rate constant, *k*_*FRET*_ (Bunt and Wouters, 2017). This can be represented as an effectively short donor–acceptor distance, even though the actual distances in an oligomer may be longer depending on the complex architecture. For computational tractability, we approximated the oligomeric *k*_*FRET*_ with a short distance and a narrow distribution (*σ*_*ol*_ = 1 Å).

Fitting was performed in two rounds: first the dimer, *R*_*di*_, and oligomer distance, *R*_*ol*_, were allowed to vary freely (Figure 3D; Supplementary Figures S2B,C), and then with distances fixed to the average values obtained in round 1 to stabilize the fits (Supplementary Figure S5D). For MC4R-A we obtained an average *R*_*di*_ of 60.0 ± 7.2 Å (mean ± SD) and *R*_*ol*_ of 37.4 ± 3.2 Å, and for MC4R-B2 we obtained an average *R*_*di*_ of 60.1 ± 7.5 Å and *R*_*ol*_ of 37.3 ± 4.2 Å, i.e., both MC4R-A and MC4R-B2 show identical inter-fluorophore distances. Finally, we plotted the resulting species fractions of the fixed distances model - monomer, dimer, and oligomer - as a function of total protein concentration, color-coded by acceptor expression (Figure 3E; Supplementary Figures S5E–J). As expected, the monomer fraction decreased with increasing concentration, whereas the dimer fraction increased. The oligomer fraction increased modestly but consistently, reaching a maximum of 20% for both variants.

Taken together, these results indicate that, in addition to robust dimerization, both MC4R-A and MC4R-B2 form a minor population of higher-order oligomers in live cells.

### 3.4 Segmentation extends concentration ranges in live-cell experiments

Using a titration series with varying D to A ratios, 1:1 to 1:20, we probed receptor concentrations up to ∼50 μM, with acceptor concentrations reaching ∼22 µM. However, at donor-to-acceptor ratios of 1:10 or higher, it became increasingly difficult to identify cells that expressed both donor- and acceptor-tagged constructs. Notably, proteins in living cells are typically not evenly distributed; instead, they accumulate locally in specific organelles or, in the case of GPCRs, concentrate in endocytic or recycling vesicles (Eichel and von Zastrow, 2018) or membrane nanodomains (Calebiro and Grimes, 2020; Calebiro et al., 2021). These heterogeneous concentration patterns can be leveraged into an analytical advantage, as they naturally extend the accessible concentration range and reduce intracellular averaging, thereby enhancing the discriminative power for quantifying monomers, dimers, and higher-order oligomers.

**FIGURE 4:**
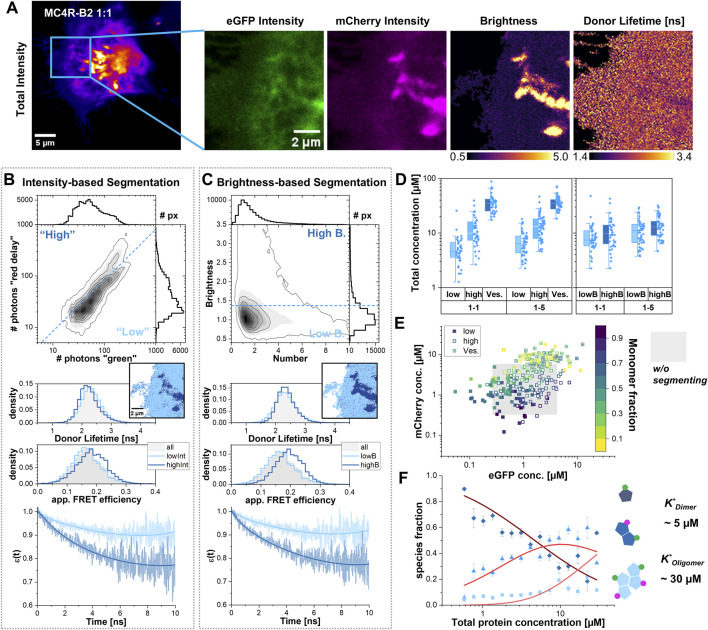
Intensity and brightness-based cell segmentation expands the accessible concentration range by intra-cell heterogeneities illustrated for MC4R-B2. **(A)** Donor fluorescence lifetimes, brightness and intensity images in HEK293T basal plasma-membranes co-transfected with MC4R-B2-eGFP and mCherry highlight heterogeneities (green: eGFP fluorescence intensity, magenta: directly excited mCherry intensity) **(B)** Intensity-based segmentation: 2D pixel-histograms of the green and red-delay photon counts with marginal distributions. Pixels above the dashed line were assigned as “high” and pixels below the line as “low” intensity region. The middle panel shows the pixel-wise mean donor fluorescence lifetime and intensity-based apparent FRET efficiency (light grey) for the whole cell, the low-intensity regions (light blue), and high-intensity regions (dark blue). The bottom panel shows the corresponding FRET-induced donor decay decays, ε(t), for the high and low intensity regions. **(C)** Same as **(B)** for the brightness-based segmentation with a threshold level of *B* = 1.375. **(D)** Total concentrations up to 100 µM were obtained after segmenting each cell into three intensity regions (low, high, vesicle) or the brightness regions (lowB, highB). **(E)** Donor vs. acceptor concentration for the intensity-based sub-segmentation (low: filled symbol; high: open symbol, vesicle: crossed symbol), color-coded by the fraction of monomers (from global analysis). The grey-shaded area indicates the concentration range probed without the sub-segmentation. The segmentation procedure broadens the concentration range. **(F)** Average monomer (dark blue), dimer (blue), and oligomer (light blue) fractions vs*.* total protein concentrations. The protein concentrations were grouped into logarithmically spaced bins. The corresponding species fractions were fitted with the oligomerization model (dark red, red. and light red, Supplementary Equations S19–S21). Reported apparent association constants, $K_{D}^{*}$ , are mean values, 95% confidence interval range from 3.76–7.20 µM and 15.3–35.0 µM, respectively. The error bars are standard errors of the means. MC4R-A results are shown in Supplementary Figure S8.

To exploit this spatial heterogeneity, we applied two sub-segmentation approaches. Since fluorescence distributions and underlying biological structures vary between systems and experimental conditions, the optimal segmentation strategy cannot be defined *a priori*; we therefore systematically evaluate two different strategies here (Figure 4A; Supplementary Figure S6). The first approach is intensity-based, while the second relies on the Number&Brightness (N&B) method (Digman, et al., 2008) (Supplementary Figure S7), which evaluates pixel-wise fluorescence fluctuations across the acquired image series (50 frames per measurement). Segmenting the images extends the concentration range and reduces the number of measurements.

In our intensity-based segmentation strategy, demonstrated for an MC4R-B2 cell, we construct pixel-wise 2D histograms of green/donor photon counts versus red photons detected after direct acceptor excitation (“red delay”) (Figure 4B, top) which are divided into “low” and “high” regions. The “high” region emits more acceptor photons compared to the “low” region. As expected, the “high” region shows shorter donor fluorescence lifetimes, *τ*, and higher intensity-based apparent FRET efficiencies, *E*_*app*_, in pixel-wise distributions (Figure 4B, middle). Likewise, the increase of FRET in “high” regions is highlighted by the time-dependent depopulation of the donor’s excited state in the FRET-induced donor decay *ε*(t) (Peulen, et al., 2017) (Figure 4B, bottom). In an *ε*(t) analysis, the donor decay in the presence of an acceptor, *f*_*DA*_ *(t)*, is divided by the reference donor-only decay, *f*_*DO*_ *(t)*. The slope of *ε*(t) reflects the FRET-rate constant, while its offset corresponds to the fraction of non-interacting molecules *x*_*noFRET*_ (monomer/unfavorable oriented dimer dipoles). In the “high” region, *x*_*noFRET*_ decreased to ∼0.75 compared to ∼0.9 in the “low” region, confirming the increased proportion of interacting receptors. In our fully automated intensity-based analysis workflow, we first discriminated rapidly moving vesicles, which contain densely packed receptors, and treated them as separate pixel class (Supplementary Figure S6). The remaining membrane region was segmented automatically into “high” and “low” intensity subregions using Otsu thresholding; and donor fluorescence intensity decays were extracted for each.

In the second N&B-based segmentation strategy, we compute the intensity mean and variance over pixels. The apparent brightness *B* is the ratio of variance to mean intensity. The apparent number *N* is the total intensity divided by *B* (Digman, et al., 2008). Monomeric fluorophores exhibit *B* ≈ 1 (Supplementary Figure S4). A 2D *N* vs. *B* histogram (Figure 4C, top) reveals distinct populations corresponding to different molecular brightness. Pixels with *B* > 1.375 were classified as “highB”, while all others were assigned to “lowB”. As expected, “highB” pixels were enriched in regions containing a larger fraction of dimeric or oligomeric receptors, consistent with the trends observed in the intensity-based segmentation (Figure 4C, middle/bottom).

Fluorescence intensities and donor decays were exported for the five different pixel categories - “low” intensity, “high” intensity, “vesicles” (very high intensity), “lowB”, and “highB” (brightness) - from D:A samples transfected at 1:1 and 1:5 ratios. We calculated the corresponding protein concentrations (Figure 4D; Supplementary Figure S8A). As anticipated, “high” and “vesicles” regions contained elevated protein levels relative to “low”, whereas “lowB” and “highB” showed only small differences in concentration. Depending on the biological question, vesicles may be of particular interest - for example, in studies of GPCR activation or trafficking - or may be excluded. In the subsequent analysis, we included vesicles as high-concentration regions. All subregions were analyzed using the previously established three-state model (monomer-dimer-oligomer) with fixed dimer and apparent oligomer donor-acceptor distances. Donor versus acceptor concentrations were plotted and color-coded by monomer fraction (Figure 4E). For the intensity-based segmentation of MC4R-B2, a clear decrease in monomer fraction was observed in high concentration regions, accompanied by increased dimer and oligomer fractions (Supplementary Figures S5B–F). Importantly, the intensity-based segmentation extended the accessible concentration range and ratios - indicated by the grey rectangle in Figure 4E and Supplementary Figure S8 - thus facilitating *K*_*D*_ estimation without requiring extreme transfection ratios (and reducing the number of required live-cell experiments).

To estimate affinity constants, the species fractions were grouped by total protein concentration into 15 logarithmically spaced bins and fitted using an oligomerization model (Figure 4F; Supplementary Equations S19–S21) to yield $K_{Dimer}^{*}$ of ∼5 µM (95% confidence interval: 3.8–7.2 µM) for MC4R-B2 and ∼6 µM (5.0–7.8 µM) for MC4R-A (Supplementary Figure S8G; Supplementary Table S5). These values are apparent association constants, as the analysis is based on FRET-active donor–acceptor complexes only (the full justification is provided in Section 4.2). The $K_{Oligomer}^{*}$ for both variants exceeded the measured concentration range (>30 μM, 9–60 µM). Note that fitting the non-binned (Supplementary Figures S5H,I) or the full ROI data (Supplementary Figure S9) results in similar values (Supplementary Table S6). In contrast, while the N&B-based segmentation successfully identified pixels with elevated apparent brightness (“highB”), the resulting expansion of accessible protein concentration range was relatively small compared to the intensity-based segmentation (Supplementary Figure S10). In this particular example the N&B approach provided only a limited additional leverage for estimating oligomerization equilibria, although it offers a distinct advantage in differentiating concentration from axial membrane position effects, as it relies on intensity fluctuations rather than absolute count rates. The intensity-based segmentation produced a substantially wider distribution of protein concentrations, thereby improving the robustness of the *K*_*D*_ estimation. Nonetheless, N&B may be more powerful in systems with larger brightness heterogeneity more strongly reflecting underlying oligomeric states. Nevertheless, comparing these approaches allows us to identify consistent trends across segmentation strategies and increases confidence that the observed effects are not artifacts of a particular analysis workflow.

### 3.5 Identifying interactions with single fluorescent protein tags

So far, we focused on FRET between spectrally distinct fluorophores (heteroFRET). Energy transfer between identical fluorophores - a process known as homoFRET - can be used in combination with heteroFRET to determine whether protein A interacts exclusively with itself (homoFRET) or additionally with protein B (heteroFRET). In contrast to heteroFRET, homoFRET does not affect the fluorescence lifetime of the donor; instead, it manifests as a reduction in fluorescence anisotropy (Liput et al., 2020; Vogel, et al., 2015; Vogel, et al., 2009). Fluorescence anisotropy compares the difference between parallel and perpendicular emission intensities following excitation with linearly polarized light. The resulting steady-state anisotropy, *r*_*ss*_ (based on fluorescence intensities, Supplementary Equation S12), and time-resolved anisotropy, *r*(*t*), (based on the time-resolved fluorescence decays) reflects the rotational mobility of the fluorophore. Rapidly tumbling molecules exhibit greater depolarization and thus lower anisotropy values. Fluorescent proteins in solution typically have steady-state anisotropies around ∼0.33, while fluorescent proteins fused to membrane proteins - such as GPCRs - exhibit higher values due to restricted rotational diffusion.

Like the heteroFRET analysis, the homoFRET workflow proceeds stepwise: (*i*) Determine the apparent (uncorrected) steady-state anisotropy per cell and plot it against concentration or count rate, (*ii*) determine polarization-dependent correction factors for the instrument, (*iii*) examine the time-resolved fluorescence anisotropy decays to identify fast components, and (*iv*) fit the time-resolved anisotropy decays using appropriate rotational or energy-transfer models.

Steady-state anisotropy images (Supplementary Figure S11) visually highlight qualitative differences between dim and bright cells (expression level). Steady-state anisotropies depend on the tumbling of the fluorophores that can be affected by their local environment. Freely and bound rotating FPs with a lifetime of 2.4 ns and rotation times of 16 ns and 100 ns have steady-state anisotropies of 0.33 and 0.37, respectively. Notably, we find a higher anisotropy for membrane anchored FPs in MC4R-A compared to non-membrane anchored FPs in MC4R-B2 (Figure 1A).

**FIGURE 5:**
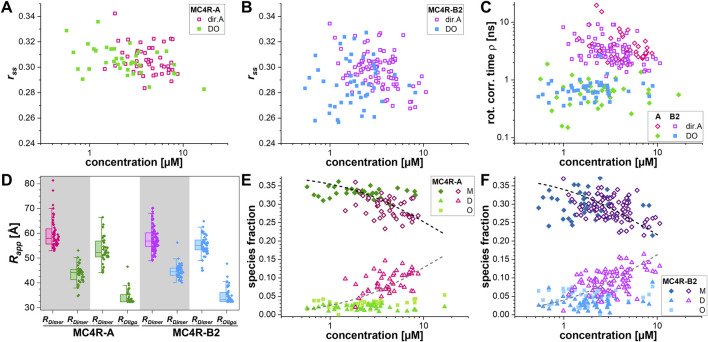
Single-color experiments: HomoFRET detects dimerization and oligomerization. **(A)** Steady-state anisotropy of eGFP in the absence of acceptors (DO, green) and directly excited acceptor mCherry (direct Acc., magenta) in MC4R-A transfected cells. Concentrations resemble the total protein concentrations. **(B)** Similar experiment for MC4R-B2 (DO cells in blue; co-transfected cells in violet). **(C)** A dimer model (see Supplementary Equation S7) was used to estimate rotational correlation times *ρ* for the cells in **(A)** and **(B)**. **(D)** Apparent distance between fluorophores (*R*_*app*_), estimated from *ρ* using a dimer model (grey) in MC4R-A (left) and MC4R-B2 (right). Magenta and violet indicate direct acceptor excitation, while green and blue show DO. An oligomer model (Supplementary Equation S8, white background) was required to fit the MC4R-A/B2 DO data. **(E)** Estimated species fractions from globally fixed FRET-rate constants in MC4R-A-eGFP (green shades) and directly excited acceptors (dir.A) in MC4R-A-mCherry (magenta shades, dimer model). For DO samples, oligomer and dimer fractions are grouped (triangles), and a dimerization isotherm is fitted to the joint data of DO and dir.A (top: black for monomer, bottom: grey for dimer). Abbreviations: M = monomer, D = dimer, O = oligomer. **(F)** Same as **(E)** for the MC4R-B2 variant.

A quantitative analysis of absolute anisotropies requires careful attention to the instrument’s beam path and rigorous sensitivity calibrations. HomoFRET experiments are fundamentally incompatible with differential interference contrast (DIC) microscopy, as the latter relies on manipulating the polarization state of light. Furthermore, any optical components that introduce additional depolarization, such as high-NA objectives, must be carefully identified and corrected to ensure the accuracy of the measured anisotropy. Upon polarized excitation, homoFRET introduces an additional depolarization pathway, reducing the observed anisotropy. For accurate analysis, the additional depolarization in high-NA objectives must be quantified and corrected (Supplementary Figure S12; Supp. Methods) (Erdelyi, et al., 2014; Koshioka, et al., 1995; Liput, et al., 2020). We quantified the steady-state anisotropy $r_{\text{ss}}$ (Supplementary Equation S12) for both DO samples and for the directly excited acceptor signal (“red delay”) in the D:A-transfected cells (1:1 and 1:5; Figures 5A,B). Please note that the concentration refers to the total protein concentration (D + A) as the monomer-dimer-oligomer equilibrium is also influenced by the (invisible) donor-tagged receptors in the same cell (For reference, Supplementary Figures S13A,B shows the same data using only the acceptor concentration). At comparable concentrations, MC4R-B2-eGFP exhibited lower $r_{\text{ss}}$ values and weaker concentration dependence than MC4R-A-eGFP. For mCherry, the anisotropy depended strongly on acceptor concentration, again with slightly lower values for MC4R-B2 compared to MC4R-A at matched expression levels (Figures 5A,B).

To jointly interpret heteroFRET and homoFRET of eGFP and mCherry data, differences in the Förster radius, $R_{0}$ , must be considered, which define the accessible distance range. For eGFP-mCherry, eGFP-eGFP, and mCherry-mCherry $R_{0}$ are 52 Å, 50 Å, and 44 Å, respectively (Lambert, 2019). In the FRET-sensitive distance range of roughly $0.5–1.5\;R_{0}$ (Hellenkamp et al., 2018), we probe distances up to ∼67 Å for an mCherry-mCherry and up to ∼78 Å for an eGFP-mCherry pair. The effective FRET-rate constant is doubled in homoFRET due to the bidirectional energy transfer (Liput, et al., 2020). Consequently, in oligomeric states, lifetimes can fall below the instrument response function width, typically 200–500 ps. A qualitative indicator for fast depolarization is a drop of the initial anisotropy decay amplitude, $r_{0}$ , using the anisotropy decay of an FP control ( $r_{0}$ ≈ 0.38) as a reference. Fast depolarization processes such as homoFRET reduce *r*_0_ (Supplementary Figure S12F) and reflect in steady-state anisotropy images and time-resolved anisotropy decays for three MC4R-A-eGFP cells (Supplementary Figure S11A). For MC4R-A, we find a correlation between a steady-state anisotropy reduction and a drop in *r*_0_ compared to the eGFP references. We observed a similar trend for MC4R-B2-mCherry in co-transfected eGFP and mCherry cells (Supplementary Figure S11B). Both are a clear qualitative indicator for MC4R oligomerization.

### 3.6 Quantifying interactions with single fluorescent protein tags

We process the homoFRET data independently of the heteroFRET data, not using knowledge of the oligomerization to compare the outcomes of the two methods. We analyze time-resolved anisotropy homoFRET data using a dimer and an oligomer model, as *a priori*, it is unclear whether a biomolecule dimerizes or forms oligomers. Analogous to the heteroFRET approach, we performed the analysis in two rounds. In the first round, samples were analyzed individually to obtain an average homoFRET-rate constant (Supplementary Figures S13C–L). In the second round, to stabilize the analysis, species amplitudes were the sole variable parameters. Our monomer model has one relaxation time for the global rotation, while dimer and oligomer mixture models have 2 and 3 relaxation times. Membrane proteins rotate slowly compared to the fluorescence lifetime (Balakrishnan et al., 2022). Thus, the rotational component was fixed to 100 ns.

When analyzing the data using dimer models in the first round (individual analysis of samples), we observed an increase of the fast relaxation amplitude and a drop in the relaxation time from ∼10 ns to ∼1 ns (Figure 5C) along with increasing acceptor concentrations. Surprisingly, in DO samples, the fast relaxation time remained nearly constant (∼0.8 ns) (Figure 5C). The concentration-independent behavior for eGFP was unexpected. A close inspection of the model parameters revealed that the perpendicular channel contained substantial short-lived “scatter” components (Supplementary Figures S13E,G). For mCherry the scatter contribution was considerably lower and showed a linear relationship between parallel and perpendicular channels (Supplementary Figures S13F,H). These observations suggested a missing anisotropy amplitude (as seen in Supplementary Figures S11, S12) was compensated/masked by “scatter”. Therefore, we reanalyzed both eGFP and mCherry data of DO and co-transfected datasets, respectively, by our oligomer model (Supplementary Figures S13I–L). In the oligomer model, oligomers result in a drop of the time-resolved fluorescence anisotropy at short times. Thus, instead of being described by scattered light, eGFP data is characterized by oligomer formation (Supplementary Figures S13E,G). For mCherry, contrary to eGFP, we found low oligomer fractions (Supplementary Figures S13J,L), likely due to the shorter *R*_0_ of mCherry.

To compare heteroFRET and homoFRET, the obtained relaxation times were converted to FRET-rate constants and apparent distances (Supplementary Equations S7–S11; Figure 5D). In the dimer model, we obtained average distances of ∼60 Å for MC4R-A-mCherry ( ρ ∼4.32 ns), ∼44 Å for MC4R-A-eGFP ( ρ ∼0.74 ns), ∼57 Å for MC4R-B2-mCherry ( ρ ∼3.33 ns), and ∼45 Å for MC4R-B2-eGFP ( ρ ∼0.72 ns). The oligomer model for MC4R-A-eGFP yielded distances of 53 Å ( $\rho_{di}$ ∼3.09 ns, dimer) and 34 Å ( $\rho_{ol}$ ∼0.22 ns, oligomer), while MC4R-B2 produced 55 Å ( $\rho_{di}$ ∼2.58 ns) and 35 Å ( $\rho_{ol}$ ∼0.19 ns), respectively. For the oligomer model, the apparent distances match the heteroFRET-derived distances, whereas the dimer distances appeared shorter compared to the heteroFRET distances. To further compare homo- and heteroFRET, we determined species fractions to estimate homoFRET affinity constants (Figures 5E,F). The pooled eGFP and mCherry data show a clear decrease of monomers with a concomitant increase in dimers/oligomers. A dimer model fitted to the pooled data of MC4R-A and MC4R-B2 estimates $K_{Dimer,pooled}^{*}$ of 26 µM and 15 μM, respectively (Supplementary Table S7). Consistent with the heteroFRET data, we find a higher dimerization affinity for MC4R-B2. This demonstrates how homoFRET can complement heteroFRET experiments.

### 3.7 Homo- and HeteroFRET occur in parallel in the co-transfected samples

**FIGURE 6:**
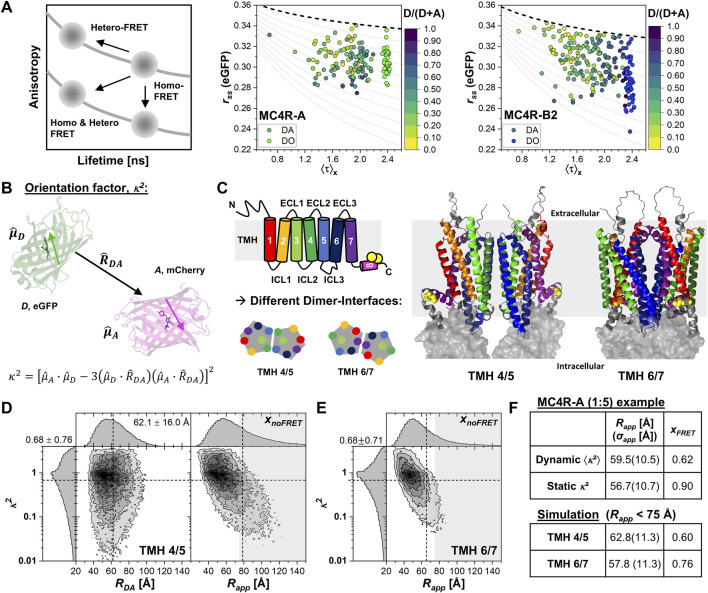
HomoFRET and heteroFRET occur simultaneously and simulation-guided analysis can help defining the interaction interface. **(A)** (left) Average fluorescence lifetime and observed steady-state anisotropies are related by the Perrin-equation (grey, $r_{obs}/r_{0}=1+\tau \rho^{-1}$ ). HeteroFRET reduces the donor fluorescence lifetime and increases the anisotropy. HomoFRET reduces anisotropy without altering the fluorescence lifetime. The scatter plot combe average lifetimes and steady-state anisotropies of MC4R-A (middle) and MC4R-B2 (right). Co-transfected cells are color-coded by stoichiometry ([D]/[D + A]). DO samples appear in light green (MC4R-A) and blue (MC4R-B2). Black and grey lines show the bi-exponential Perrin-equation [ $r\left(\tau \right)/\;r_{0}=x{\left(1+\tau \left(\rho_{Dimer}^{-1}+k_{FRET}\right)\right)}^{-1}+\left(1-x\right){\left(1+\tau \rho_{Mc4r}^{-1}\right)}^{-1}$ ]. Here, x is the fraction of a fast component (increasing from 20%–65% in 5% steps), and $k_{FRET}$ = 0.62 ns^-1^ (i.e., the average FRET-rate of the dimer model). **(B)** The orientation factor *κ* ^2^ depends on the mutual donor and acceptor dipole orientation defined by the unit vectors ${\hat{\mu }}_{D}$ , ${\hat{\mu }}_{A,}$ and ${\hat{R}}_{DA}$ . **(C)** AlphaFold3 (Abramson, et al., 2024; Jumper et al., 2021) predicts multiple dimerization interfaces for MC4R-A. ICL: Intracellular loop; ECL: Extracellular loop; TMH: Trans-membrane helices; N: N-terminus, C: C-terminus; yellow circles: membrane anchor. Dimerization occurs via TMH 4/5 (left) or TMH 6/7 (right). eGFP and mCherry are shown as semitransparent surface. Grey-shaded area is the cell membrane. The TMHs are rainbow-colored from red (helix 1) to purple (helix 7), with the cytoplasmic helix in pink. The same color code was used for the dimerization interfaces. **(D)** We simulated inter-fluorophore distances, *R*_*DA*_, orientation factors *κ* ^2^, and resulting apparent distances *R*_*app*_ of FPs attached to MC4R-A for the TMH4/5 dimerization interface shown in **(C)**. Dashed lines mark mean values for *κ* ^*2*^ *, R*_*DA,*_ and *R*_*app*_. The 2D histogram shows the mean and width of the distributions. **(E)** Simulated apparent inter-fluorophore distances for the TMH6/7 dimerization model. **(F)** (Top) We analyzed MC4R-A cells with a donor-to-acceptor ratio (D:A) of 1:5 = ?), using a Gaussian model to measure distances (Supplementary Equations S4–S6). The orientation factor *κ* ^2^ was set to a dynamic value of 2/3 and assumed to be isotropically distributed (static). (bottom) Mean and width values are shown for the dimer models (TMH4/5 and TMH6/7). Models with a measured distance *R*_*app*_ > 75 Å are labeled as *x*_*noFRET*_. A detailed simulation workflow and details on dimer models TMH1/7 and TMH3/4, see Supplementary Figure S14.

We processed homo- and heteroFRET data independently; however, as dimers and higher-order oligomers form stochastically, both homo- and heteroFRET occur in parallel. HomoFRET affects the fluorescence anisotropy while heteroFRET primarily shortens the donor lifetime (Figure 6A). To visualize the simultaneous contribution of homo- and heteroFRET in our samples, we plotted steady-state anisotropies against the donor fluorescence lifetime for all DA and DO cells. As expected, the formation of dimers and higher-order oligomers led to concurrent changes in fluorescence lifetime and anisotropy: heteroFRET shortens the donor lifetime, homoFRET reduces the observed anisotropy.

When visualizing experiments in a two-dimensional anisotropy-lifetime space, as expected, only a set of Perrin curves ( $r_{obs}=r_{0}/\left(1+\frac{\tau }{\rho }\right)$ can describe the data, as the downward shift driven by homoFRET and an upward/leftward shift driven by heteroFRET-induced lifetime reductions happen simultaneously to a different extent (Figure 6A). However, within the data clear systematic trends are observed for both MC4R-A and MC4R-B2, highlighted by a color-coding of the stoichiometry shown as the ratio of donor to total protein concentration (Figure 6A, middle/right). With decreasing donor ratio, the DA samples shift towards shorter lifetimes and lower anisotropy values, a hallmark for homo- and heteroFRET. To guide the visualization, we have added a set of Perrin lines for slow rotating molecules with varying degrees of homoFRET. With decreasing donor fraction, the data points shift to Perrin lines with a higher fraction of fast depolarization (top black line: 20%, bottom grey line: 65%), confirming that both processes occur in parallel in the cellular environment.

Although in the present experiments both eGFP and mCherry were fused to the same receptor variant, the observed spread of points in the anisotropy-lifetime space illustrates the sensitivity of this approach to mixed interaction modes: In co-expression experiments of distinct receptor variants (e.g., MC4R-A-eGFP with MC4R-B2-mCherry), the relative contribution of homotypic (A-A, B2-B2) versus heterotypic (A-B2) interactions could be estimated as a function of the respective donor and acceptor concentrations.

### 3.8 Accounting for orientational κ^2^ effects

The observed FRET efficiency, E , is determined by three main factors: (*i*) the inter-fluorophore distance $R_{DA}$ , (*ii*) the spectral overlap between donor emission and acceptor excitation (determining the Förster radius together with donor fluorescence quantum yield), and (*iii*) the relative orientation of the donor and acceptor transition dipole moments, *κ* ^2^ (Förster, 1948) (Figure 6B). Notably, the influence of *κ* ^2^ is often neglected as it is experimentally challenging to determine (Hummer and Szabo, 2017; Meer et al., 2013). Two limiting regimes can be distinguished: in the dynamic averaging regime, fluorophores rotate fast compared to their fluorescence lifetime (rotation ≪ τ), allowing their orientations to average (Dale et al., 1979; Peulen, et al., 2017). In the static regime fluorophores rotate slowly compared to the fluorescence lifetime and thus requires considering a *κ* ^2^ distribution (Hummer and Szabo, 2017; Meer, et al., 2013; Peulen, et al., 2017). For intensity-based FRET, an average $\langle \kappa^{2}\rangle$ of 0.476 should be used (Meer, et al., 2013). FPs attached to a protein can be sterically restrained. Thus, fluorescence analysis benefits from molecular simulations to assess spatial and *κ* ^2^ distributions.

As an example, we simulate the *κ* ^*2*^ distribution of a heteroFRET pair by feeding the sequences of MC4R-A-eGFP and MC4R-A-mCherry into Alphafold3 (AF3) (Abramson, et al., 2024) and build the dimer models (Evans et al., 2022). AF3 predicts models with four distinct dimerization interfaces. Dimerization occurred either via transmembrane helix (TMH) 4/5, TMH6/7, TMH5/6 or TMH3/4 (Figure 6C). Notably, for GPCRs, all these different interaction interfaces have been found (Breitwieser, 2004). To obtain distributions over inter FP distances and orientation factors we coarse grain the MC4R-FP dimers in the integrative modelling platform (IMP) (Russel, et al., 2012) and sample the sterically allowed conformational space by Markov-Chain-Monte-Carlo (Supplementary Figure S14). For the obtained models, we compute inter-fluorophore distances, *R*_*DA*_ and *R*_*app*_, and orientation factors (Figures 6C–E; Supplementary Figure S14). The approach to coarse grain a sample conformational spaces in IMP was implemented in open-source software, FPSIMP (https://github.com/fluorescence-tools/fpsimp) While in all models the average ⟨*κ* ^2^⟩ only slightly deviated from 2/3, the broad *κ* ^2^ distribution highlights its influence on *R*_*app*_ (Figures 6D,E; Supplementary Figure S14).

Even though one may expect steric restraints imposed by the linkage and the membrane, we find a good agreement between the peak positions of apparent distance, *R*_*app*_, as compared to the distance distribution, *R*_*DA*_ (Figure 6D). This agreement supports our analysis by a simple distribution model, which does not explicitly account for the orientation factor distribution. Note, *R*_*DA*_, is the physical distance, while *R*_*app*_ is an apparent distance that originates from the assumption that ⟨*κ* ^2^⟩ = 2/3. The main discerning feature between *R*_*DA*_ and *R*_*app*_ distributions is the tail towards longer apparent distances. A direct fluorescence decay analysis of MC4R-A 1:5 transfected cell by a single Gaussian distribution (Supplementary Equations S3–S5) in the dynamic and static regime (Figure 6F) supports this finding: the inter-fluorophore distances change marginally from 60 Å to 57 Å while *x*_*FRET*_ increases from 62% to 90%. The difference in the fraction of FRET-active species is explained by the explicit handling of “missing/invisible” fraction of FRET-molecules due to unfavorably oriented dipoles. While fast rotating fluorophores adopt conformations favorable for FRET, slowly rotating fluorophores are “stationary”. Thus, orientational factors can prevent energy transfer, even at short distances. Accordingly, our definition of $x_{\text{noFRET}}$ throughout the study included both monomers and dimers/oligomers with unfavorable dipole orientations. Consequently, while fitting with *κ* ^2^ distributions could slightly shift the estimated *K*_*D*_, the overall interpretation of inter-fluorophore distances and oligomerization trends remains robust.

### 3.9 Live-cell FRET experiments inform on dimerization interfaces

The main objective in the design of the MC4R variants was to quantify the different dimerization degrees. Motivated by the agreement between the simulations and the experiments, we use the FRET data to study dimerization interfaces. Clearly, only given the FRET data alone, this is an underdetermined problem. However, given the FRET data and different competing models, these models can be ranked by their agreement with the experiments.

For a rigorous comparison with defined statistics, the fluorescence decays and the models are compared directly considering the data shot-noise and other experimental uncertainties (Greife, et al., 2016). In this analysis, we compare only the FRET-active species with *R*_*app*_ < 75 Å and assign all other structural models to $x_{\text{noFRET}}$ . Next, we compute average apparent distances, $\langle R_{app}\rangle$ , and distribution widths. For TMH4/5 we obtain an average of 63 Å with a width of 11 Å. The other three competing models TMH6/7 (Figure 6E), TMH1/7 (including helix 8), and TMH3/4 (Supplementary Figures S14B,C) show all very similar distance distributions of the FRET-active fraction with mean and width of 58 ± 11 Å, 57 ± 12 Å, and 59 ± 12 Å, respectively. Notably, point mutations in ICL2 and adjacent TMH3/4 region strongly reduce the dimerization (Piechowski, et al., 2013). Thus, the TMH 4/5 dimer simulations, in which the ICL2 and adjacent cytosolic TMH3 residues form the dimerization interface, agree best with our experiments (Figure 6D). In the TMH3/4 model ICL2 and intracellular residues of TMH3 are far apart. This shows that a combination of biochemical studies, live-cell FRET experiments, and structural simulations can delineate receptor arrangements and inform on dimerization interfaces.

## 4 Discussion

### 4.1 PIE-FLIM as a powerful tool to study protein–protein interactions in live cells

Disentangling the intricate interactions of biomacromolecules in living cells remains challenging and requires methods that provide nanoscale spatial sensitivity and quantitative spectroscopic readouts while remaining compatible with live-cell imaging. In this context, live-cell PIE-FLIM proved to be a particularly powerful approach. FRET combined with FLIM provides a uniquely powerful framework to study nanoscale protein-protein interactions under near-physiological conditions. By reporting on molecular proximities below the diffraction limit, these techniques are ideally suited for investigating dynamic receptor organization, structural plasticity, and state-dependent oligomerization in living cells (Greife, et al., 2016; Kravets, et al., 2016; Weidtkamp-Peters, et al., 2009; Zhang, et al., 2025). For complex assemblies such as GPCR signaling platforms, where ligand-induced rearrangements and environment-driven interactions constantly remodel the nanoscale landscape, PIE-FLIM enables quantitative and time-resolved interrogation of molecular architecture (Greife, et al., 2016; Kravets, et al., 2016; Somssich et al., 2015; Weidtkamp-Peters, et al., 2009). For GPCRs such as MC4R, ligand binding can shift the equilibrium of oligomeric states, rearrange protomer interfaces, and alter both inter- and intramolecular FRET signatures (Latorraca et al., 2017).

### 4.2 Both MC4R-A and MC4R-B2 show modest but significant oligomerization

Here we demonstrated the use of live-cell spectroscopy by re-examining the homotypic protein interactions of MC4R. Previous work has established that MC4R forms dimers (Kleinau, et al., 2020; Liu, et al., 2023; Nickolls and Maki, 2006) and proposed possible interaction interfaces (Piechowski, et al., 2013), our data extends this findings by demonstrating that MC4R populates a broader spectrum of oligomeric states, ranging from monomers to higher-order assemblies. This was suggested previously (Chapman and Findlay, 2013), and we now provide quantitative estimates of the corresponding affinity constants, moving beyond the largely qualitative or binary interaction assessments reported so far.

Both MC4R-A and MC4R-B2 displayed modest but reproducible oligomerization, with MC4R-B2 consistently exhibiting a slightly higher apparent affinity than MC4R-A. This trend was independently supported by both homoFRET and heteroFRET-based analysis, lending confidence to the robustness of the observed differences. The moderate interaction strengths observed here are consistent with a dynamic equilibrium between monomeric and oligomeric receptor populations, as expected for GPCRs operating in the crowded and heterogeneous environment of the plasma membrane. Note, $K_{Dimer}^{*}$ values reflect apparent affinities under overexpression conditions; at endogenous receptor densities, particularly in hypothalamic neurons, the monomer fraction is expected to dominate. Whether agonist binding shifts this equilibrium, as proposed for other class A GPCRs (Milligan, et al., 2019), remains an important open question that future ligand-dependent FLIM-FRET experiments could address. Beyond its biophysical interest, characterization of GPCR oligomeric states has a broad biological importance: oligomerization governs receptor trafficking, alters ligand binding, and modulates pharmacological efficacy. Our quantitative spectroscopy and modeling provide a mechanistic framework linking the oligomeric status to receptor function in both physiological and pathophysiological contexts.

The two receptor variants differ in the architecture of their fluorescent protein fusions: MC4R-A contains a membrane anchor in helix VIII followed by a short flexible linker to the fluorescent protein, whereas MC4R-B2 lacks this anchor but features a longer intracellular extension preceding the tag. Because linker length, flexibility, and anchoring can in principle influence fluorophore separation and orientation, these constructs also serve as an internal methodological control for potential linker-dependent biases in FRET-based distance and interaction estimates. Notably, we did not observe systematic differences in apparent distances or FRET efficiencies between MC4R-A and MC4R-B2 attributable to linker architecture. This suggests that, within the resolution and sensitivity of our measurements, linker composition did not measurably distort the inferred interaction parameters. Nevertheless, linker structure and fluorophore mobility remain important considerations for quantitative FRET studies. A systematic investigation of linker length and sequence effects across standardized constructs would be valuable for establishing broadly transferable design rules and minimizing the need for extensive construct-specific simulations in future studies (Basak et al., 2021). The current construct design with the C-terminal FP tagging limits the ability to differentiate competing structural models. Strategically placed FPs (for instance in the N-terminus, or an intra- or extracellular loop) may increase our ability to resolve distinct dimerization interfaces.

The association constants reported here carry uncertainties arising from assumptions of the underlying models: The stepwise oligomerization model, Gaussian distance approximation, and the κ^2^ = 2/3 assumption are each reasonable but simplified. As shown in Section 3.8, an explicit κ^2^ distribution changes the FRET-active fraction substantially (62% vs. 90%) while only marginally affecting distances (60 Å vs. 57 Å), confirming that oligomerization trends are robust to this assumption. Bootstrap-derived 95% confidence intervals (Supplementary Tables S5–S7) quantify remaining parameter uncertainty. Future studies employing position-specific FP labeling or single-molecule approaches could further constrain these assumptions.

Both heteroFRET and homoFRET individually provide only a partial view of the oligomerization equilibrium: heteroFRET detects only mixed donor–acceptor complexes, while donor–donor and acceptor–acceptor species remain invisible yet still participate in the same equilibrium. As a result, the extracted dissociation constants are reported as apparent *K*_*D*_ values based on a simplified binding isotherm. However, when donor- and acceptor-tagged receptors distribute randomly within oligomeric assemblies, as expected for stochastic co-transfection, the fractions of DD, DA/AD, and AA complexes follow binomial statistics, and heteroFRET- and homoFRET-derived association constants are expected to converge (Greife, et al., 2016). The observed agreement, apparent $K_{Dimer}^{*}$ of ∼5 µM from heteroFRET and $K_{Dimer,pooled}^{*}$ of ∼15–26 µM from homoFRET, is consistent with this expectation given the different Förster radii of the two readouts, and we consider this convergence as corroborating evidence for the robustness of the reported equilibria. A fully integrated homo/heteroFRET framework that explicitly accounts for all complex species remains an important direction for future work.

### 4.3 Challenges in live-cell FLIM and spectroscopic measurements

Accurate analysis is a central prerequisite for the correct interpretation of the acquired imaging data. Prior to this study, the lack of user-friendly, open-source analysis workflows presented a major obstacle. Many laboratories rely on closed vendor platforms or custom scripts written in MATLAB, which hinders reproducibility and accessibility. In response, our step-by-step described workflow provides a fully open-source, beginner-friendly toolkit composed of GUI-driven applications and Jupyter notebooks (Greife, et al., 2025). This approach not only lowers the barrier for new users but also maximizes transparency and reproducibility of PIE-FLIM data analysis. A further central prerequisite for quantitative FRET measurements is that the fluorescent protein (FP) fusion does not alter the biological behavior of the protein of interest. For the constructs used in this study, receptor functionality was confirmed by cAMP signaling assays (Liu, et al., 2023) and cellular localization was verified by 3D confocal imaging of fixed cells (Supplementary Figure S1). The agreement between homoFRET and heteroFRET-derived oligomerization parameters provides an additional internal consistency check, as two independent spectroscopic readouts sensitive to different aspects of receptor proximity yield concordant *K*_*D*_ estimates. A further important distinction concerns apparent FRET-derived distances versus structural distances. The inter-fluorophore distances recovered in this study are apparent distances *R*_*app*_, derived under the assumption of κ^2^ = 2/3. They reflect both the physical fluorophore separation *R*_*DA*_ and the orientational averaging of the transition dipoles. As shown in Figures 6D,E, the peak of the *R*_*app*_ distribution aligns well with the peak of the *R*_*DA*_ distribution for all tested dimerization models, supporting the use of *R*_*app*_ as a reliable proxy for structural proximity. However, the tail of the *R*_*app*_ distribution extends to longer distances relative to *R*_*DA*_, as a direct consequence of orientationally unfavorable dipole configurations. Readers should therefore interpret the recovered distances as apparent proximity indicators rather than precise structural measurements. Absolute structural distances require either explicit κ^2^ distributions from molecular simulations, as demonstrated here using IMP, or independent constraints from complementary structural data. Additionally, tags must be positioned within a FRET-accessible range; *R*_*0*_ values range ∼30–69 Å for common FP pairs listed in FPbase (Lambert, 2019), thus giving an accessible range of up to 110 Å.

Moreover, accurate quantification of protein-protein interactions in living cells is inherently challenged by spatial heterogeneity in protein distribution, local concentration, and cellular context. In earlier FLIM studies regions of interest were selected manually, e.g., membrane-associated receptor populations or specific intracellular compartments (Kravets, et al., 2016). Such manual region selection can be subjective, difficult to reproduce, and scales poorly to large datasets. Here, we demonstrate an automated PIE-FLIM image segmentation analysis that can improve robustness and information yield by subdividing cells into biologically meaningful subregions. Our approach analyzes biomolecular states as a function of the local environment and protein density. Such spatial stratification reduces averaging, enhances sensitivity to intracellular heterogeneity, and expands the effective concentration range, central for the reliable determination of oligomerization constants. An integrated analysis workflow enables the incorporation of image- and spectroscopy-based features. Local intensity distributions, molecular density proxies, shape descriptors, and N&B–derived estimates can be coupled to fluorescence lifetime, anisotropy, and FRET efficiency measurements. Linking these observables allows improving the discrimination between monomeric, dimeric, and higher-order assemblies. By shifting from manually curated regions to reproducible, feature-based spatial analysis, segmentation becomes a key component of quantitative live-cell interaction studies rather than a purely technical preprocessing step.

Overexpression may increase local receptor densities beyond physiological levels, potentially shifting the equilibrium toward higher-order assemblies - a limitation inherent to transient transfection systems. In our experiments, we observed intracellular vesicle-like structures (Supplementary Figure S1), which are identified as fast-moving pixel classes and treated separately in the segmentation workflow (Figure 4; Section 3.4). Excluding or including these high-density compartments allows the user to assess their contribution to the measured equilibrium. Importantly, the concentration-dependent analysis presented here explicitly accounts for expression level variation by using per-cell fluorescence intensities rather than transfection ratios, and by spanning a wide concentration range through both titration experiments and intra-cell segmentation. Note that absolute concentration estimates depend on system-specific calibration parameters (e.g., detection volume and molecular brightness), which must be determined individually for each microscope setup. The consistency of apparent *K*_*D*_ values obtained from full-ROI and segmented sub-ROI analyses (Supplementary Tables S5, S6) further supports the robustness of the reported equilibrium constants against expression heterogeneity. At the same time, elevated receptor densities can promote bystander FRET and shift apparent equilibria away from physiological conditions. While our concentration-dependent analysis partially mitigates this, such effects cannot be entirely excluded in overexpression systems. Alternative strategies - including tunable promoters or knock-out cell lines with controlled re-expression - can reduce these artifacts and will be important when applying this framework to other receptor systems where endogenous expression is non-negligible.

## 5 Outlook

We demonstrated how to disentangle oligomerization states in living cells using homo- and heteroFRET. Nevertheless, live-cell spectroscopy remains challenging due to constraints in sample preparation and data analysis. High-quality fluorescence lifetime imaging requires careful microscope calibration, control of imaging conditions, and limiting phototoxicity/photobleaching due to high irradiances. Likewise, quantitative interpretation of FRET signals is complicated by the need to disentangle photophysics, labeling stoichiometry, dipole orientations, and receptor dynamics (Wallrabe et al., 2006). Advances in FP engineering - including brighter, photostable variants (e.g., mStayGold, mScarlet (Gadella et al., 2023; Goedhart and Gadella, 2024) and red-shifted donor-acceptor combinations (Zhang et al., 2023) - have helped mitigate phototoxicity and improve live-cell compatibility by lowering the excitation density and required FP overexpression for detectability.

Still, the efficient use of the limited photon budget motivates approaches that maximize the information extracted from every photon by combining homo- and heteroFRET. Generally, homoFRET provides a unique advantage: it requires only a single fluorophore species, eliminating chromatic aberrations and freeing a second spectral channel for independent biological markers such as biosensors of cell state, second messengers, or receptor activity. This is especially valuable in live-cell imaging, where phototoxicity, acquisition speed, and multiplexing capacity are often limiting (Liput, et al., 2020; Wallrabe, et al., 2006). The combination of fluorescence anisotropy and fluorescence lifetime allows simultaneous visualization of homo- and heteroFRET in parallel when studying homotypic and heterotypic protein interactions (Liput, et al., 2020). Although a unified lifetime-anisotropy framework that jointly describes all acquired data is not yet available, we demonstrated consistent results for homo- and heteroFRET data acquired by pulsed-interleaved excitation (Muller, et al., 2005) with multiparameter polarization resolved detection (Weidtkamp-Peters, et al., 2009) which simultaneously records hetero- and homoFRET data. Such combination is particularly well suited in GPCRs to dissect also state-dependent interactions with downstream signaling proteins and identifying conformational coupling within receptor complexes (Harkes et al., 2021). Mapping anisotropy versus lifetime provides a two-dimensional representation that separates homoFRET-induced depolarization from heteroFRET-driven lifetime shortening. This integrated representation not only unifies both FRET readouts but also enables approximation of relative interaction contributions, extending analytical power beyond what homo- or heteroFRet alone can achieve.

Fluorescent proteins can only provide limited structural information. However, we demonstrated how limited information can be useful to falsify competing structural models by combining structural prediction tools such as AlphaFold (Abramson, et al., 2024) or RoseTTAFold (Baek et al., 2021) with integrative coarse-grained approaches for multi-scale simulations in the Integrative Modeling Platform (Russel, et al., 2012). Central to relate structures to spectroscopic FRET observables, particularly in fluorescent proteins, is the handling of linker regions and the orientation factor *κ* ^2^. A persistent challenge in quantitative FRET analysis is the uncertainty which reflects the relative orientation of donor and acceptor transition dipoles. For fluorescent proteins, rotational diffusion is slow relative to their fluorescence lifetime, rendering the commonly assumed isotropic average of *κ* ^2^ = 2/3 generally invalid (Hummer and Szabo, 2017; Meer, et al., 2013; Peulen, et al., 2017). As a result, absolute distance and interaction estimates derived from FRET measurements may be systematically biased if dipole orientation constraints are not considered. We demonstrated how *κ* ^2^ distributions, the spatial freedom, steric constraints, and preferred orientations of fluorescent protein tags within the molecular assembly can be handled *in silico*. A direct experimental access to these distributions is limited, and brute-force molecular dynamics simulations remain computationally demanding for large, membrane-embedded protein–fluorophore systems. Thus, future iterative refinement between structural modeling and experimental fluorescence data offers a promising strategy to reduce uncertainty in *κ* ^2^ and to improve the quantitative interpretation of interaction parameters for calibrated experiments and simulations. Beyond improving affinity estimates, such integrative approaches also provide a structural framework for understanding receptor organization and conformational coupling within oligomeric assemblies.

## Data Availability

The datasets presented in this study can be found in online repositories. The names of the repository/repositories and accession number(s) can be found below: https://doi.org/10.5281/zenodo.17869432 https://doi.org/10.5281/zenodo.18175059.
