# Supplementary material for: Bound or unbound: mapping and monitoring receptor oligomerization by time-resolved fluorescence live-cell imaging
Source: Front Mol Biosci. 2026 Jun 16;13:1814859. doi: 10.3389/fmolb.2026.1814859 (PMC13314469; doi:10.3389/fmolb.2026.1814859)
Supplement: Supplementary file 1 [file DataSheet1.pdf]

Supplementary Material

Bound or unbound: Mapping and monitoring receptor oligomerization by time-resolved fluorescence live-cell imaging

Annemarie Greife[1#], Ruiqi Liu[2#], Paul S. Köhler[3], Katrin G. Heinze[3], Katherina Hemmen[3*], Thomas-Otavio Peulen[3,4*]

Supplementary Figure 1. Volumetric imaging of HEK293T cells co-transfected with eGFP- and mCherry-tagged MC4R shows membrane localization of MC4R. 9

Supplementary Figure 2. Fluorescence lifetime images of representative cells 11

Supplementary Figure 3. Fit model selection for DO lifetime models and DA Gaussian Distance models. 12

Supplementary Figure 4. Estimating receptor concentration based on Fluorescence Correlation Spectroscopy (FCS). 13

Supplementary Figure 5. Concentration-dependent changes of MC4R monomer, dimer, and oligomer fractions. 14

Supplementary Figure 6. Subcellular analysis of the cell ROIs. 15

Supplementary Figure 7. Number & Brightness approach. 16

Supplementary Figure 8. Fit results for the intensity-based sub-segmentation for MC4R-A and MC4R-B2. 17

Supplementary Figure 9. Oligomerization model for MC4R-A and MC4R-B2 full cell ROIs. 18

Supplementary Figure 10. Results of the Oligomerization model for the MC4R-A and MC4R-B2 brightness-based sub-segmentation. 19

Supplementary Figure 11. Steady-state fluorescence anisotropy images of selected cells. 20

Supplementary Figure 12. Characterization of the depolarization through the microscope objective. 21

Supplementary Figure 13. Comparison of dimer and oligomer models in time-resolved anisotropy measurements. 22

Supplementary Figure 14. Simulation workflow to estimate the spatial fluorescent proteins distribution on target proteins and resulting FRET efficiencies for protein complexes. 23

Supplementary Table 1.. 25
Supplementary Table 2. 26
Supplementary Table 3. 26
Supplementary Table 4. 26
Supplementary Table 5.. 27
Supplementary Table 6. 27
Supplementary Table 7. 27

1 Supplementary Methods: Detailed Data Analysis

1.1 Live-cell Fluorescence Lifetime Imaging (FLIM)

Export of intensity images. Fluorescence intensity images were generated by integrating photons of all frames in the three specified channels: green-prompt (donor emission), red-prompt (FRET-sensitized acceptor emission), and red-delay (directly excited acceptor emission). The data export into 16-bit tiff-files was programmed in scripts using *tttrlib* (Peulen et al. 2025) and *scikit-image* (van der Walt et al. 2014).

Image segmentation. To define cells and regions of interest (ROIs) for each acquired image, the total intensity sum of all three channels (green prompt, red prompt and red delay) was used. The image of the intensity sum was in a first step smoothed by using a median filter with a two-pixel radius, followed by Li thresholding as implemented in *scikit-image* (Li and Lee 1993, Li and Tam 1998). In the next step, holes smaller than 200 pixels were filled and small objects (e.g. cells at the image border) encompassing less than 10'000 pixels removed. The binary images, *i.e.* the cell masks, were exported as 8-bit tiff files with intensity values = 0 inside the cells and 255 outside the cell. The automated segmentations were controlled and curated in Fiji (Schindelin et al. 2012).

Sub-region segmentation. Within the cell membrane the fluorescence intensity was inhomogeneous. To assess if intensity variations and dimerization differences are related, ROIs were segmented into five different sub-ROIs using two distinct methods. In the first method, the vesicles detected mainly in the red channels were framewise selected in the red delay time series using an *ilastik* based segmentation (Berg et al. 2019). In *ilastik*, a pixel classifier is trained based on user input to recognize structures of interest. We used the cell density counting approach implemented in *ilastik* trained on manually annotated examples from representative images to generate pixel-wise vesicle probability maps (sigma=2.50) and exported binary cell masks. In the next step, the green prompt time series were loaded jointly with the vesicles mask and the cell mask. All pixels belonging to the vesicles were removed from the cell mask and the obtained cell mask was applied onto the green prompt intensity images to build frame integrated intensity images (sum image). The sum image intensity histogram was used to split cells into a "low" and a "high" intensity region by Otsu's thresholding implemented in *scikit-image* (Otsu 1979). In the second method, we used the "Number & Brightness" (N&B) approach (Digman et al. 2008), that relies on the intensity fluctuations along the image time series to elucidate an pixel-wise brightness and apparent number of molecules. We compute intensity fluctuations based on the total intensity in all channels (green prompt, red prompt and red delay) and split cells in a "low B" (brightness below 1.375, assumed to be rather monomeric) and a "high B" brightness region (brightness above 1.375, assumed to be rather dimeric). The segmented five different regions were converted into binary masks, saved as 8-bit tiffs, and used to export the fluorescence intensity decays and other properties of these regions.

Export of fluorescence intensity decays. The exported binary ROI images from the image segmentation were used as selective mask to compute the fluorescence decay histograms of the ROI using *tttrlib* and *scikit-image*. The fluorescence decay histograms were computed for all channels (green prompt, red prompt and red delay) with a twofold micro time binning resulting in a histogram bin size of 20 ps. The signal from the parallel and perpendicular detection channel was stacked into a single column.

Determination of correction factors. FLIM measurements were performed with vertical polarized excitation (V) and detection, either in plane (VV) or 90° rotated (VH) to the excitation direction. The used high NA objectives lead to polarization mixing that needs to be considered to compute artifact

free fluorescence intensity decays (Erdelyi et al. 2014, Koshioka et al. 1995). These polarization mixing factors are denoted as l_1 (crosstalk from perpendicular into parallel channel) and l_2 (crosstalk from parallel into perpendicular channel). Moreover, the detection sensitivities of the parallel and perpendicular detectors need to be corrected by the g-factor, g, typically defined as the sensitivity ratio of the parallel (I_{VV}) and perpendicular (I_{VH}) detector. In an ideal system, g is close to 1. A g-factor of two means that the detection system is twice as sensitive to vertically polarized light as it is to horizontally polarized light. To determine g, l_1, and l_2 we jointly analyze the fast rotating fluorophores Alexa488 and Alexa568 along with slowly rotating fluorescent proteins (FPs) in *ChiSurf* (Peulen 2025) using a single rotational correlation time and mono- (Alexa dyes) or bi-exponential fluorescence lifetime models (FPs). Details on the fitting procedure can be found in the software manual. On average, the green g-factor was 0.946 with l_1 and l_2 being 0.059 and 0.305, respectively. The red g-factor was 0.995, and l_1 and l_2 were 0.121 and 0.279, respectively.

Determination of average fluorescence lifetimes. Average fluorescence lifetimes of eGFP-tagged receptor transfected (DO) and eGFP- and mCherry-tagged receptors co-transfected FRET samples (DA) for the eGFP fluorescence channel (488 nm excitation) were determined in *ChiSurf* by fitting by iterative reconvolution of multi-exponential models to the exported time-resolved fluorescence intensities:

$$f_D(t) = \sum_m x_m \exp\left(-\frac{t}{\tau_m}\right) \qquad \text{with } \sum_m x_m = 1 \qquad \text{eq. S1}$$

Briefly, experimental nuisances were introduced into the model by convolutions with the IRF, computing the partial polarization mixing, and scaling models by the detection sensitivity of the detectors with m=2 for DO samples and m=3 for FRET samples. Thus,

$$I(t) = N_0 \cdot f_D(t) \otimes IRF + sc \cdot IRF + B \qquad \text{eq. S2}$$

Here, *sc* is due to scattered light from the sample, and *B* the constant offset. The model intensity decay histograms, $I(t)$, were scaled to the experimental measured number of photons to reduce the number of free fitting parameters (the initial amplitude N_0 is not fitted). For the multiexponential model, the average fluorescence lifetime $\langle\tau\rangle_x$ was calculated as species-average:

$$\langle\tau\rangle_x = \sum x_m \tau_m \qquad \text{eq. S3}$$

Determination of inter-fluorophore distances (heteroFRET). Inter-fluorophore distance distributions in FRET samples were determined by fitting a mixture model $f(t)$ to the data, which contains the fluorescence decay of non-FRET molecules, $f_{DO}(t)$, and $f_{DA}(t) = \epsilon_D(t) \cdot f_{DO}(t)$, the fluorescence decay of FRET species:

$$f(t) = (1 - x_{FRET}) \cdot f_{DO}(t) + x_{FRET} \cdot (f_{DO}(t) \cdot \epsilon_D(t)) \qquad \text{eq. S4}$$

Here, x_{FRET} is the fraction of FRET active species and $\epsilon_D(t)$ is the FRET-induced donor decay that can be directly related to FRET rate constants and distances (Peulen et al. 2017).

Due to the flexible C-terminal tagging of the FPs, we relate the FRET-induced donor decay to a distance distribution. In this analysis, use a Gaussian distribution of half-width σ_{app} and centered around $\bar{R}_{app}$. In this analysis, we compute FRET-rate constants for distances for a single averaged κ^2 of 2/3, yielding apparent inter-fluorophore distances; potential deviations due to restricted fluorophore

orientations are addressed in the **Discussion**. Accordingly, the recovered distances represent apparent distances, R_{app}, that reflect both spatial separation and orientational averaging rather than absolute fluorophore separation. Nevertheless, these recovered distances directly relate to the FRET-induced donor decay $\epsilon_D(t)$ by:

$$\epsilon_D(t) = \int p(R_{app}) \exp\left(-\frac{t}{\tau_0} \cdot [R_0/R_{app}]^6\right) dR_{app} \,, \qquad \text{eq. S5}$$

where the inter-fluorophore distribution $p(R_{app})$ is approximated by a normal distribution:

$$p(R_{app}, \sigma_{app}, \overline{R_{app}}) = \frac{1}{2\pi\sigma_{app}^2} \exp\left(-\frac{1}{2}\left[\frac{R_{app}-\bar{R}_{app}}{\sigma_{app}}\right]^2\right). \qquad \text{eq. S6}$$

Here, we used a literature value of 52 Å for the used eGFP/mCherry FRET-pairs (Lambert 2019).

In the first analysis round, we fitted the fluorescence decays of entire cells by a single Gaussian $\bar{R}_{app}$ and σ_{app} (σ_{app} ranged from 5 to 25 Å). In the second analysis round, we fitted the fluorescence decays of entire cells by a mixture of two Gaussians. The longer distance, $\bar{R}_{di}$ represents the MC4R dimer distance (σ_{di} fixed to 6 Å). In the presence of multiple acceptors, all acceptors contribute to FRET (sum of quenching processes). Accordingly, a short apparent distance with a narrow distribution (σ_{app} ≈1 Å) was used as an effective description of collective quenching by multiple acceptors in oligomeric assemblies (Greife et al. 2016, Kravets et al. 2016). In the third analysis round, the average values for $\bar{R}_{di}$ and $\bar{R}_{ol}$ were fixed to determine species fractions for the entire cells and the five segmented cell ROIs with σ_{di} ranging from 5 to 25 Å. Note that σ_{di} was fitted jointly over the five sub-ROIs from each cell.

Inter-fluorophore distances determined by homoFRET. Fluorophores show fluorescence depolarization due to their rotational motion. FPs alone have a rotational correlation time, ρ_{FP}, of around 12 ns (Striker et al. 1999). For the FP coupled to MC4R we estimated that the rotational correlation is slower ($\rho_{global} \approx$ of 100 ns). The global rotational correlation time of ~100 ns we set consistent with previous measurements of eGFP-tagged GPCRs in cell membranes, where values between 75–200 ns have been reported depending on the attachment site (Balakrishnan et al. 2022). This slow rotation is characteristic of membrane-embedded proteins and produces a plateau in the time-resolved anisotropy decay. Fitting this plateau as a free parameter yields values with large uncertainties (50–10,000 ns); we therefore fixed this component to 100 ns as a physically motivated constraint. In homoFRET, a FRET-induced (additional) fluorescence depolarization of the emitted light reports on fluorophore proximity. In homoFRET the relaxation time, ρ_{FRET}, decreases with the distance, eventually lowering the initial amplitude of the fundamental anisotropy, r_0. We jointly fitted the parallel, $I_{VV}(t)$, and perpendicular, $I_{VH}(t)$, fluorescence decays using bi-exponential (dimer) and tri-exponential models (oligomer):

$$\frac{r(t)}{r_0} = (x_{mo} + x_{di}e^{-2k_{di}t})e^{-\frac{1}{\rho}t} \qquad \text{eq. S7}$$

$$\frac{r(t)}{r_0} = (x_{mo} + x_{di}e^{-2k_{di}t} + x_{ol}e^{-2k_{ol}t})e^{-\frac{1}{\rho}t} \qquad \text{eq. S8}$$

Here, ρ is the global rotational correlation time of the protein bound FP, x_{mo}, x_{di}, and x_{ol} are species fractions of the monomer, dimer, and oligomer, respectively, with corresponding FRET-rate constants k_{di} (dimer) and k_{ol} (oligomer).

In our data analysis, we described $r(t)$ by a multiexponential decay and determine relaxation times ρ_{di} and ρ_{ol}, which are coupled to the global molecule rotation, ρ:

$$\rho_{di} = \left(\frac{1}{\rho} + 2k_{di}\right)^{-1} \quad \text{and} \quad \rho_{ol} = \left(\frac{1}{\rho} + 2k_{ol}\right)^{-1} \qquad \text{eq. S9}$$

Note, in contrast to unidirectional heteroFRET, a factor of two is included in the equation as homoFRET occurs bidirectional between donor molecules. We relate the FRET-rate constants to apparent distances in the dimer, R_{di}, and oligomer, R_{ol}, using the Förster equation:

$$k_{di} = \frac{1}{\tau_0}\left(\frac{R_0}{R_{di}}\right)^6 \quad \text{and} \quad k_{ol} = \frac{1}{\tau_0}\left(\frac{R_0}{R_{ol}}\right)^6 \qquad \text{eq. S10}$$

To obtain R_{di} and R_{ol} for measured relaxation times ρ_{di} and ρ_{ol}:

$$R_{di} = R_0\left(\frac{1}{2}\left(\frac{1}{\rho_{di}} - \frac{1}{\rho}\right)\tau_0\right)^{-1/6} \quad \text{and} \quad R_{ol} = R_0\left(\frac{1}{2}\left(\frac{1}{\rho_{ol}} - \frac{1}{\rho}\right)\tau_0\right)^{-1/6}. \qquad \text{eq. S11}$$

Here, we used $R_{0,eGFP}$ = 49 Å and $\tau_{0,eGFP}$ = 2.38 ns for eGFP and $R_{0,mCherry}$ = 44 Å and $\tau_{0,mCherry}$ = 1.31 ns for mCherry (Lambert 2019). Note, analogous to heteroFRET samples, distance distributions are a more accurate description. Nevertheless, for simplicity in the analysis, we fitted discrete relaxation times, ρ, and computed corresponding FRET-rate constants.

In addition to the time-resolved anisotropies, we compute the steady-state anisotropy, r_{ss}, of eGFP in eGFP-only samples, eGFP/mCherry transfected samples and r_{ss} of directly excited mCherry using the background-corrected count rates in the parallel, S_{VV}^{BG}, and perpendicular channels, S_{VH}^{BG}.

$$r_{ss} = \frac{S_{VV}^{BG} - g \cdot S_{VH}^{BG}}{S_{VV}^{BG} + 2 \cdot g \cdot S_{VH}^{BG}} \qquad \text{eq. S12}$$

Here, the background of the channels was estimated by the fitted offset of the fluorescence decay curves in the respective detection channel.

Concentration estimation: Converting photon counts to concentration. To relate the changes in dimer or oligomer fractions to the MC4R concentration, we performed fluorescence correlation spectroscopy (FCS) on reference samples in singly transfected (MC4R-eGFP or MC4R-mCherry) cells to determine the molecular brightness (kilocounts per molecule per second, kcpms) of the fluorescent proteins. Using these experimental kcpms, the measured fluorescence intensities can be converted to the number of molecules and concentrations (Hemmen et al. 2021). FCS measurements were performed at the basal membrane and analyzed using a two-dimensional diffusion model to account for membrane-associated receptor dynamics (Balakrishnan, et al. 2022):

$$G(t_c) = b + \frac{1}{N}\left(\frac{a_1}{1+\frac{t_c}{t_{D1}}} + \frac{1-a_1}{1+\frac{t_c}{t_{D2}}}\right) \cdot (1 - a_B + a_B \cdot \exp -t_c/t_B) \qquad \text{eq. S13}$$

where b is the offset of the correlation curve $G(t_c)$, N the number of molecules in focus, a_1 the fraction of fast diffusion molecules with diffusion time t_{D1}, the slow diffusion time t_{D2} and a_B and t_B account for the photophysics/dark states fraction (a_B) and time constant (t_B).

To ensure comparability with FLIM measurements, data were acquired in single-color mode at a repetition rate of 20 MHz under conditions closely matching the FLIM acquisition settings. Background contributions were estimated from non-transfected cells and amounted to approximately 3 kHz per detector in the green channel and 1 kHz per detector in the red channel. Given the relatively low count rates and significant uncorrelated background, background correction was essential and was performed following established approaches (Thompson 2002), accounting for the reduction in correlation amplitude due to constant offset contributions.

FCS measurements in cells were conducted at varying excitation powers to find a suitable excitation regime with negligible photobleaching. In our setup we found optimal conditions at 3 µW (eGFP) and 14 µW (mCherry), resulting in average count rates of 1.0 ± 0.19 kcpms for eGFP and 0.95 ± 0.14 kcpms for mCherry. To match FLIM conditions, count rates were extrapolated to the lower excitation powers used during FLIM acquisition (0.54 µW for eGFP and 0.3 µW for mCherry). Based on our calibration curves, we assumed a linear dependence of fluorescence intensity on excitation power in this regime for eGFP. Here, the molecular brightness in solution and in the membrane were nearly identical (1.1 vs 1.0 kHz at 3 µW). Thus, we estimated ~0.18 kcpms for eGFP at our FLIM conditions of 0.54 µW. mCherry showed strongly saturating behavior and we approximated the molecular brightness at low irradiances by fitting the curve with an exponential model. This yielded 0.39 kcpms for mCherry in solution at 0.3 µW. Considering that at 14 µW mCherry in solution showed 2.9 kcpms and mCherry in the membrane 1 kcpms, we extrapolate a molecular brightness of ~ 0.13 kcpms at 0.3 µW.

Note, absolute brightness values are instrument-specific and must be determined individually for each experimental setup. The confocal microscope used in this study (LSM980) is not optimized for FCS measurements. Measurements on a dedicated, diffraction-limited FCS setup yield substantially higher molecular brightness (e.g., ~12 kcpms for Alexa488 at 1.3 kW/cm²; (Hemmen, et al. 2021)). Moreover, saturation effects need to be considered. Preferentially, measurements are performed in an excitation power regime where cpms changes are linear. For a given microscope, this can be assessed by performing a power-dependent cpms measurement series. Measurements in a linear regime ensure an unbiased estimate of the molecular brightness (Widengren et al. 1995).

The effective detection volume was determined using Alexa488 and Alexa568 reference dyes, yielding average volumes of ~0.81 fl (488 nm excitation) and ~0.92 fl (560 nm excitation), respectively. The concentration of mCherry, [A], was determined based on the background-corrected average count rate, in a ROI:

$$[A] = \frac{S^{BG}_{VV;\,red\ delay} + S^{BG}_{VH;\,red\ delay}}{brightness\ [cpms] \cdot detection\ volume\ [fl]} \qquad \text{eq. S14}$$

For the eGFP concentration in the donor only cells, $[D_0]$, the concentration estimation was performed similarly:

$$[D_0] = \frac{S^{BG}_{VV;\,green\ prompt} + S^{BG}_{VH;\,green\ prompt}}{brightness\ [cpms] \cdot detection\ volume\ [fl]} \qquad \text{eq. S15}$$

For the eGFP concentration in the FRET samples, the background-corrected average count rate was corrected for the quenching due to FRET:

$$S_{green,corr} = \frac{S^{BG}_{VV;\,green\ prompt} + S^{BG}_{VH;\,green\ prompt}}{(1-x_{FRET}) + x_{FRET}(1-E)} \qquad \text{eq. S16}$$

The energy transfer efficiency E and the fraction of the FRET-active population, x_{FRET}, were taken from the Gaussian distance fit described above (**eq. 3-5**). Subsequently, the concentration of eGFP in the presence of mCherry $[D_A]$ was estimated using the corrected count rate:

$$[D_A] = \frac{S_{green,corr}}{brightness\ [cpms] * detection\ volume\ [fL]} \qquad \text{eq. S17}$$

The ratio of the donor to the acceptor (or total protein, T) concentration influences strongly the observed amount of FRET. Thus, we define the ratio as

$$ratio\ D{:}T = \frac{[D_A]}{[D_A]+[A]} \qquad \text{eq. S18}$$

1.2 Estimation of the association constants for oligomerization.

To describe the dimerization and oligomerization of the MC4R variants, a simple stepwise model is used; in a pre-equilibrium the dimer is formed first, followed by subsequent oligomerization, similar to what was described previously (Greife, et al. 2016, Kravets, et al. 2016). In this analysis higher-order oligomers are formally represented by a tetrameric species.

$$o + o \rightarrow oo \qquad K^*_{Dimer} = \frac{[o][o]}{[oo]} \qquad \text{eq. 19}$$

$$oo + oo \rightarrow (oo)(oo) \qquad K^*_{Oligomer} = \frac{[oo][oo]}{[(oo)(oo)]} \qquad \text{eq. 20}$$

Here o is a monomer, oo a dimer and (oo)(oo) is a tetramer. We use the monomer o as the base species. Then the total protein concentration is given by:

$$c_T = [o] + 2 * [oo] + 4 * [(oo)(oo)] \qquad \text{eq. 21}$$

Now, the three species fractions x_{mo}, x_{di} and x_{ol} for any given total protein concentration are used to determine K_{Dimer} and $K_{Oligomer}$ by solving the three equations above.

1.3 Estimation of the dimerization constant from homoFRET analysis.

For the estimation of an apparent dimerization constant, $K^*_{Dimer,pooled}$, we merged the fit results from the DO and directly excited acceptor anisotropy analysis. While the acceptor signal was analysed with the dimer model (**eq. 6**), the DO samples were best described with an oligomerization model (**eq. 7**). Thus, for the DO samples we combined the species fractions x_{Di} and x_{Oligo} to yield $x_{Di,app}$. Similarly, as above, we define $K^*_{Dimer,pooled}$ and c_T, and solve the two equations:

$$o + o \rightarrow oo \qquad K^*_{Dimer,pooled} = \frac{[o][o]}{[oo]} \qquad \text{eq. 22}$$

$$c_T = [o] + 2 * [oo] \qquad \text{eq. 23}$$

1.4 Volumetric Imaging

3D volumetric images were processed in Huygens 25.04 (Scientific Volume Imaging). As a first step, the spectral crosstalk of HOECHST in the mCherry channel was removed using the Crosstalk Corrector. As a second step, the 3D stacks were deconvolved using the Deconvolution Wizard. The signal-to-noise ratio and background (0.7 µm, lowest) were automatically determined and an acuity of 0 was used. The processed stacks were exported as 16-bit tiff stacks and visualized in Imaris 11 (Bitplane, Oxford Imaging).

2 Supplementary Figures and Tables

2.1 Supplementary Figures

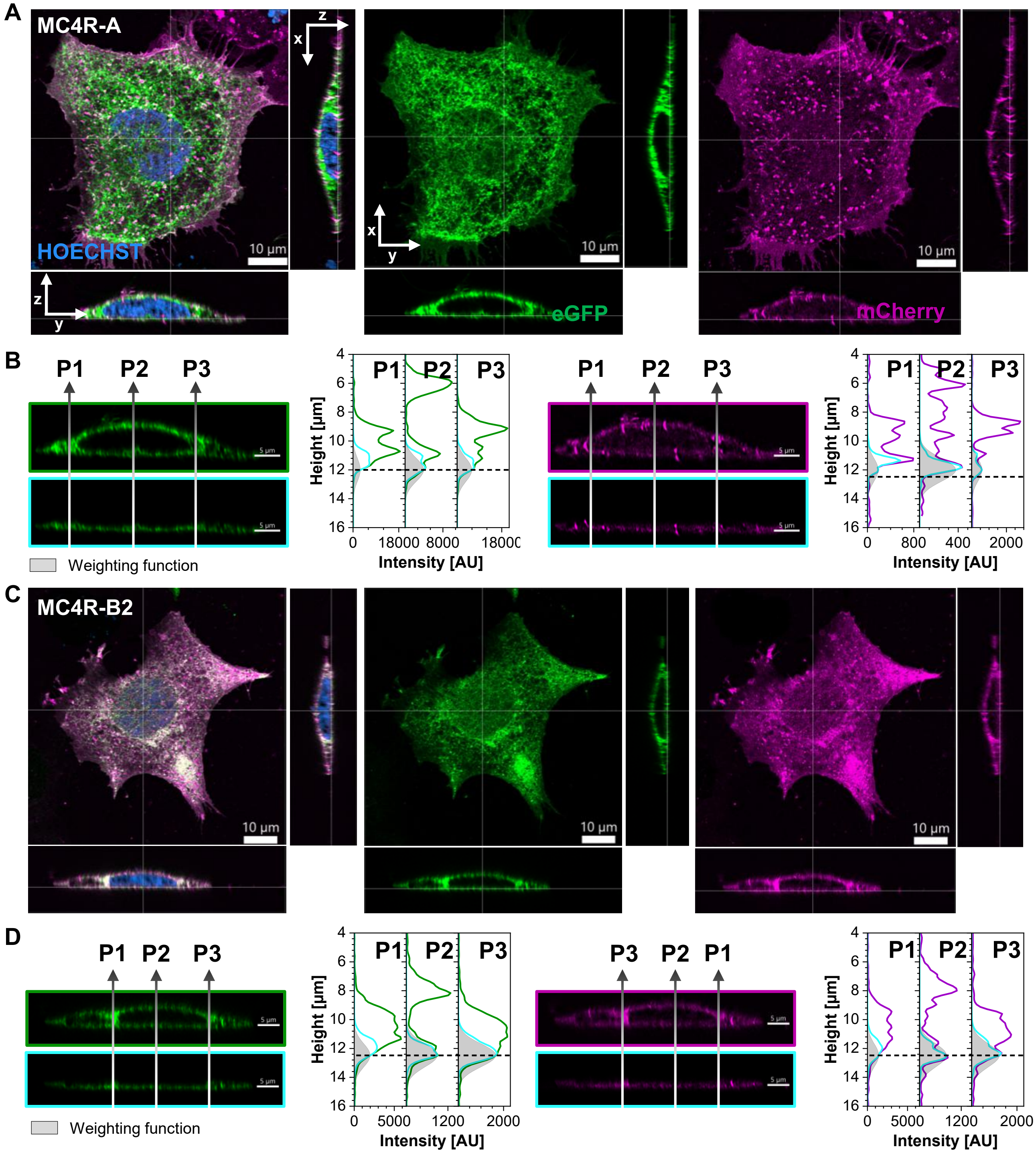

Supplementary Figure 1. Volumetric imaging of HEK293T cells co-transfected with eGFP- and mCherry-tagged MC4R shows membrane localization of MC4R. (A) Representative HEK293T cell co-transfected with eGFP- and mCherry-tagged MC4R-A. Left: overlay of eGFP (green), mCherry (magenta), and nucleus (blue) channel; middle: eGFP channel only; right: mCherry channel only. Images shown in the center are confocal slices of the basal membrane; the images shown to the right

and the bottom are projections of single x-z and y-z slices. **(B)** Out-of-focus signal visualized for the experimentally determined confocal volume. The confocal volume was determined by FCS calibration measurements. We multiplied the intensity signal with a normalized Gauss (weight at selected basal slice = 1) with a σ of 0.8 µm (average of green/red) and show the intensity along a single x-z slice before (green/magenta) and after weighting (cyan). In live-cell FLIM experiments, data was collected for a single slice in the basal membrane. The grey curve illustrates the weighting function (maximum scaled to intensity in the original data). **(C)** and **(D)** are identical for MC4R-B2. Scale bars in (A, C) are 10 µm and (B, D) 5 µm.

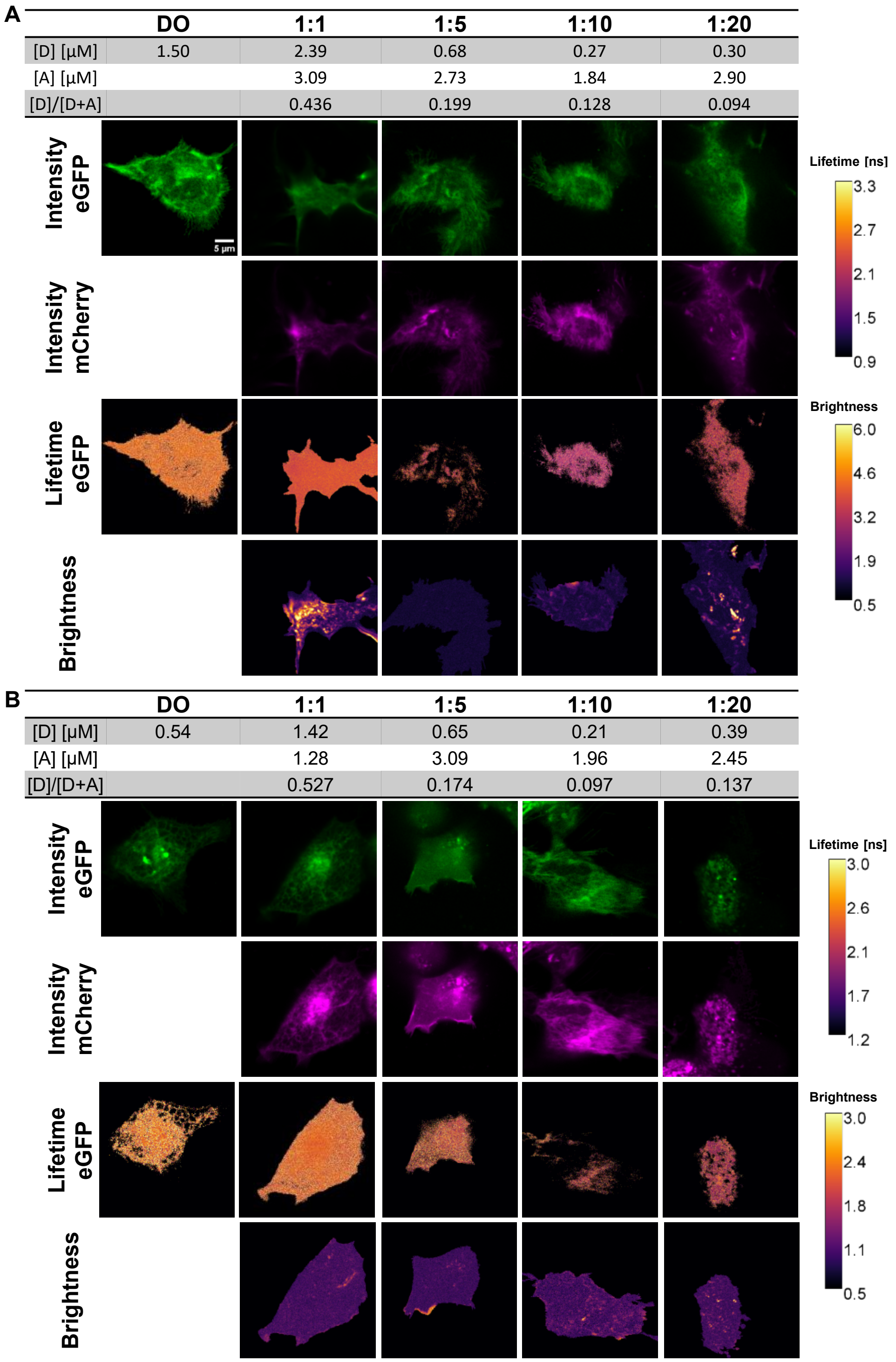

Supplementary Figure 2. Fluorescence lifetime images of representative cells (A) eGFP fluorescence intensity in the "prompt" time window (eGFP excitation, 485 nm), mCherry fluorescence intensity in the "delay" time window (mCherry excitation, 561 nm), mean lifetime and brightness images for selected MC4R-A cells either transfected with the MC4R-eGFP construct alone (DO) or in a 1:1, 1:5, 1:10 or 1:20 ratio of MC4R-A-eGFP and MC4R-A-mCherry. The total plasmid amount was kept constant. **(B)** Same as (A) for the MC4R-B2 construct. The mean lifetimes were computed for pixels with more than 15 photons. The table on top shows the donor and acceptor concentration and the concentration ratio, respectively.

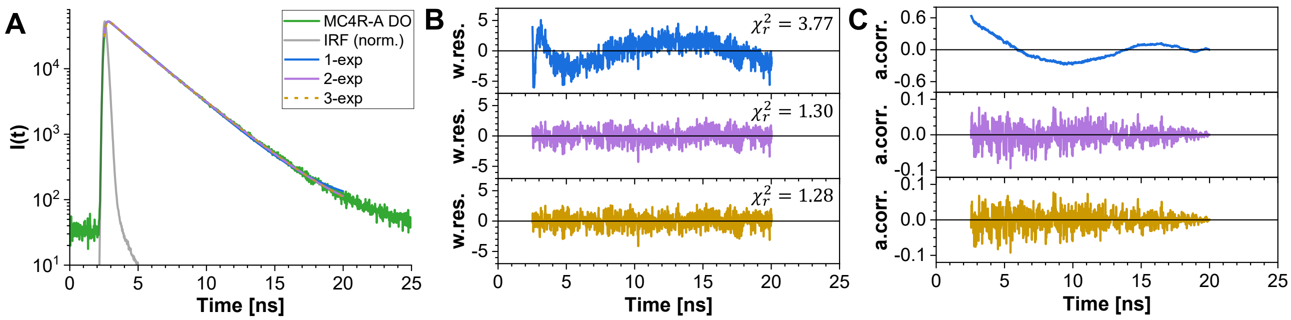

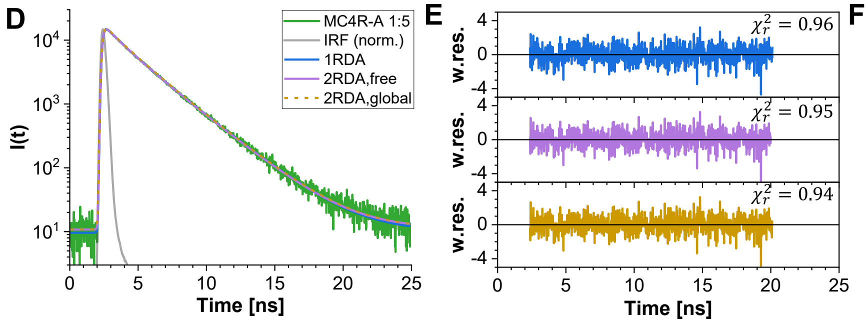

F

Model/ Parameter	1RDA	2RDA, free	2RDA, global
$R_{app,1}$ [Å] ($\sigma_{app,1}$)	55.1 (6.0)*	61.4 (6.0)*	60.4* (5.0)
$R_{app,2}$ [Å] ($\sigma_{app,2}$)	---	39.4 (1.0)*	37.5* (1.0)*
x_1 [%]	100	75.0	78.7
x_{DO} [%]	77.0	71.0	74.2

** Fixed parameter*

Supplementary Figure 3. DO lifetime and DA Gaussian Distance fit model selection. (A) Representative donor intensity decay histogram of an MC4R-A-eGFP transfected cell (green). The decay was fit by a mono-exponential (1-exp, blue), bi-exponential (2-exp, violet) or tri-exponential (3-exp, yellow) model. The Instrument Response Function (IRF) is shown in grey; its height was normalized to the data. **(B)** and **(C)** show the weighted residuals (w.res.) and autocorrelations of w.res. (a. corr.) of different fit models, respectively. Fit ranges usually begin at ~10% of the maximum intensity and cover an interval where the Poissonian data noise is approximately Gaussian distributed. To evaluate the goodness-of-fit we check if w.res. and the autocorrelation of w.res. are randomly distributed around 0. A reduced sum of weighted squared deviations, χ_r^2, close to unity indicates a good fit. Note, unaccounted noise sources may perturb the statistics. Thus, we use relative statistical tests, such as F-tests, to judge whether a model describes the data better than a reference model. Based on multiple datasets and 2-exp and 3-exp fit results, the 2-exp model describes our DO lifetimes sufficiently well. When comparing the 3-exp *vs*. 2-exp model an F-tests obtains a *p*-value of 0.437, *i.e.* no Statistical test such as F-tests can be used to judge whether the 3-exp model describes the data significantly better.t significant. **(D)** Representative donor fluorescence decay histogram of a cell co-transfected with a 1:5 of MC4R-A-eGFP and MC4R-A-mCherry (green). The decay was fitted with a single Gaussian distance (1RDA, blue), two Gaussian distances (2RDAfree, violet) or the global two Gaussian distances model (2RDAglobal, yellow). **(E)** Weighted residuals and **(F)** obtained fit parameter from the fits shown in (D). Note that when considering a single cell, all three fits would describe the data equally well (*p*-values for 2RDAfree *vs*. 1RDA model = 0.352 and 2RDAfree *vs*. 2RDAglobal model = 0.439). Here, the decision for the appropriate fit model needs to be made based on (i) the complete experimental dataset and/or (ii) (biological) pre-knowledge of the system under study. The fit models are described in **equation S1-S6** and summarized in **Supplementary Table 3**.

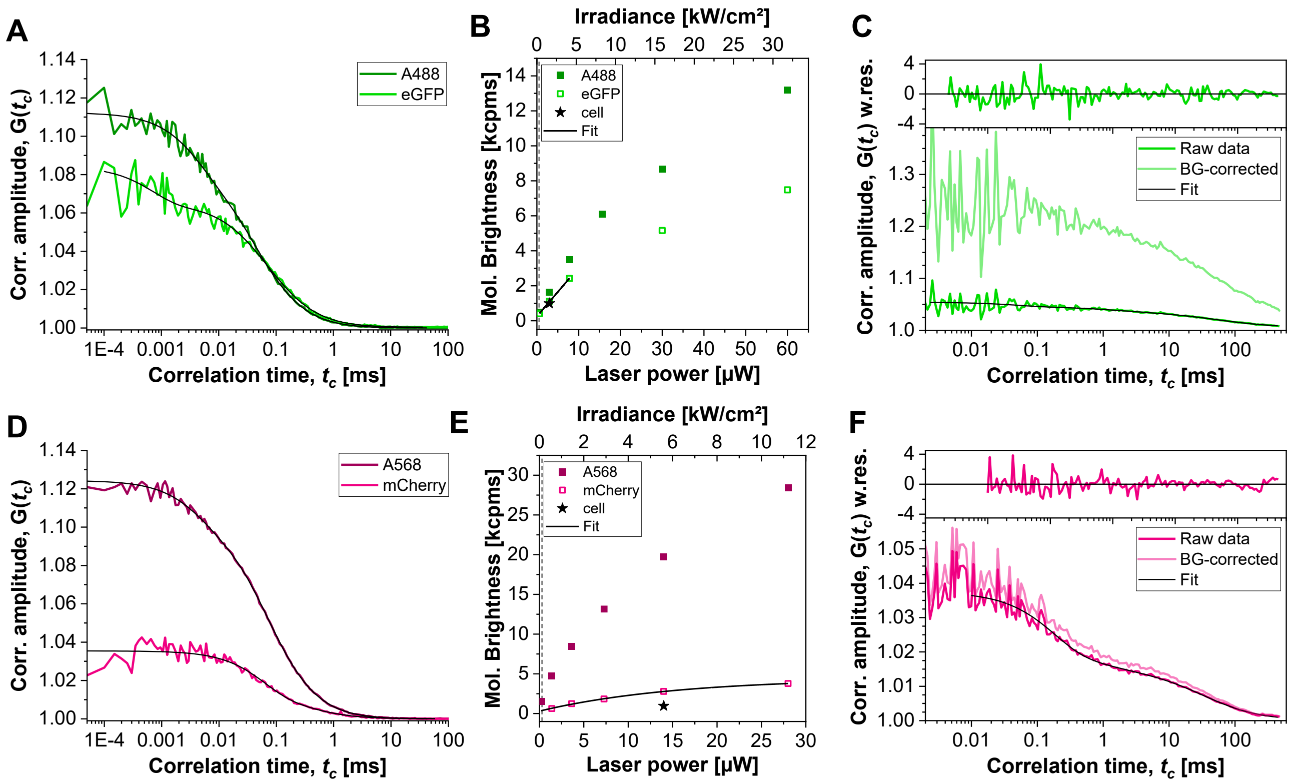

Supplementary Figure 4. Receptor concentration estimation by Fluorescence Correlation Spectroscopy (FCS). (A) FCS curves of dilute solution of Alexa488 and GFP measured at the maximal 60 µW at objective for the used setup. **(B)** Molecular brightness (kilocounts per molecule and second, kcpms) for increasing laser power at objective. Top axis indicates excitation density in kW/cm². Live-cell FLIM measurements were performed at ~0.54 µW (grey dashed line). Black star indicates average brightness of live-cell FLIM measurements. **(C)** Exemplary live-cell FCS measurement performed at 3 µW at the basal membrane of an MC4R-B2-eGFP transfected HEK293T cell (green). Auto-fluorescent cell background (~3 kHz) was corrected as described in the **Supplementary Methods** section (light green). Raw data was fit using a bimodal 2D diffusion and an additional relaxation/photophysics term including a constant background intensity correction term (black line, **eq. S13**). **(D)** FCS curves of dilute solution of Alexa488 and GFP measured at the maximal 28 µW at objective for the used setup. **(E)** Molecular brightness (kilocounts per molecule and second, kcpms) for increasing laser power at objective. Top axis indicates excitation density in kW/cm². Live-cell FLIM measurements were performed at ~0.3 µW (grey dashed line). Black star indicates average brightness of live-cell FLIM measurements, black line the exponential fit to extrapolate the brightness at low irradiances. **(F)** Exemplary live-cell FCS measurement performed at 14 µW at the basal membrane of an MC4R-A-mCherry transfected HEK293T cell (magenta). Auto-fluorescent cell background (~1 kHz) was corrected as described in the **Supplementary Methods** section (light magenta). Raw data was fit using a bimodal 2D diffusion and an additional relaxation/photophysics term including a constant background intensity correction term (black line, **eq. S13**).

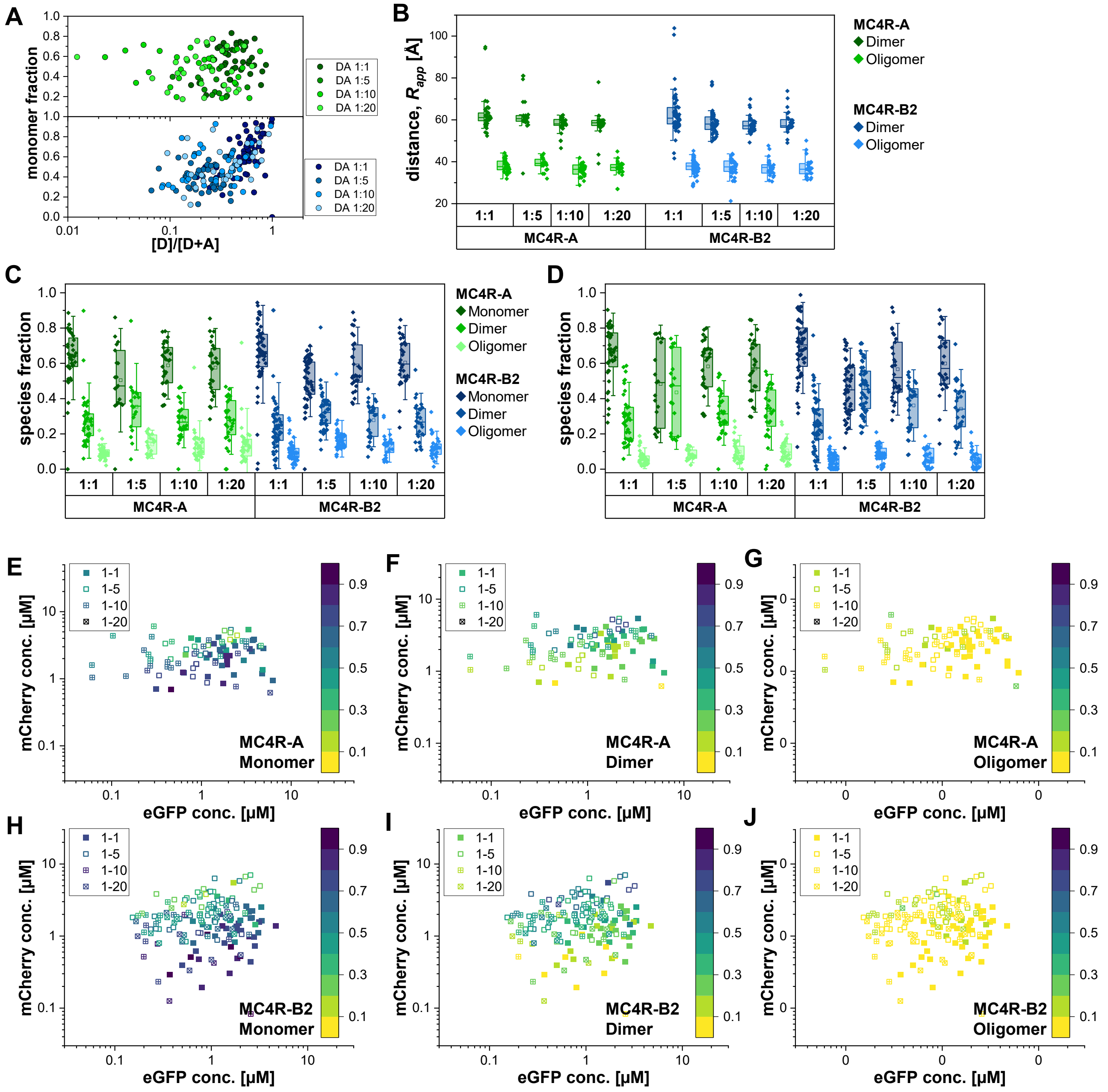

Supplementary Figure 5. Concentration-dependent changes of MC4R monomer, dimer, and oligomer fractions. (A) The monomer fractions (determined by a Gaussian model) depend on the donor to total protein concentration ratio ([D]/[D]+[A]) (MC4R-A, green; MC4R-B2, blue). **(B)** Fitted apparent mean inter-fluorophore distances for the two-Gaussian distance model. **(C)** Corresponding species fractions. **(D)** Species fraction for the **global** two-Gaussian distance model. (Dark color: monomer, mid color: dimer, lighter color: oligomer). **(E-G)** The observed species fractions depend on the donor (eGFP-tagged) and acceptor (mCherry-tagged) concentration. MC4R-A monomer (E), dimer (F), and oligomer (G) species fractions are color-coded (0 %, yellow; 100 %, dark blue). Filled, open, crossed, circles symbol corresponds to a 1:1, 1:5, 1:10, and 1:20 eGFP to mCherry transfection ratio. **(H-J)** Same as (E-G) for MC4R-B2.

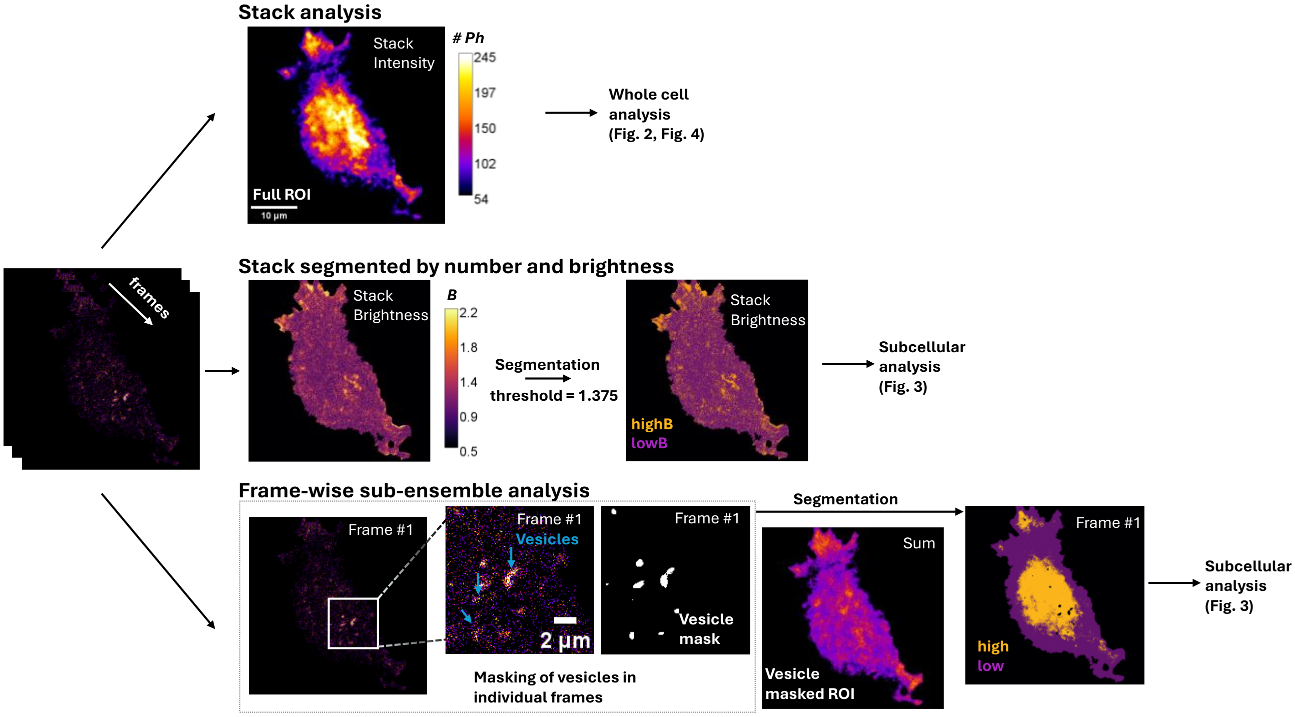

Supplementary Figure 6. Subcellular analysis of the cell ROIs. The FLIM time-series images were segmented and analyzed using three different approaches. **(Top)** In Figures 3 and 5, the whole cell was used as a region of interest (ROI). The cell outline was defined based on the stacked image. **(Mid)** In a 2nd approach, the stacked image was analyzed using the number and brightness approach, and the complete cell ROI was split into a low and high brightness region using a threshold of B =1.375. The threshold was set based on a manual inspection of multiple datasets. **(Bottom)** In the 3rd approach, high-intensity vesicles were detected on a frame-by-frame basis and removed from the cell ROI. The remaining fluorescence intensity was split frame-by-frame in a low and high intensity region using Otsu's thresholding.

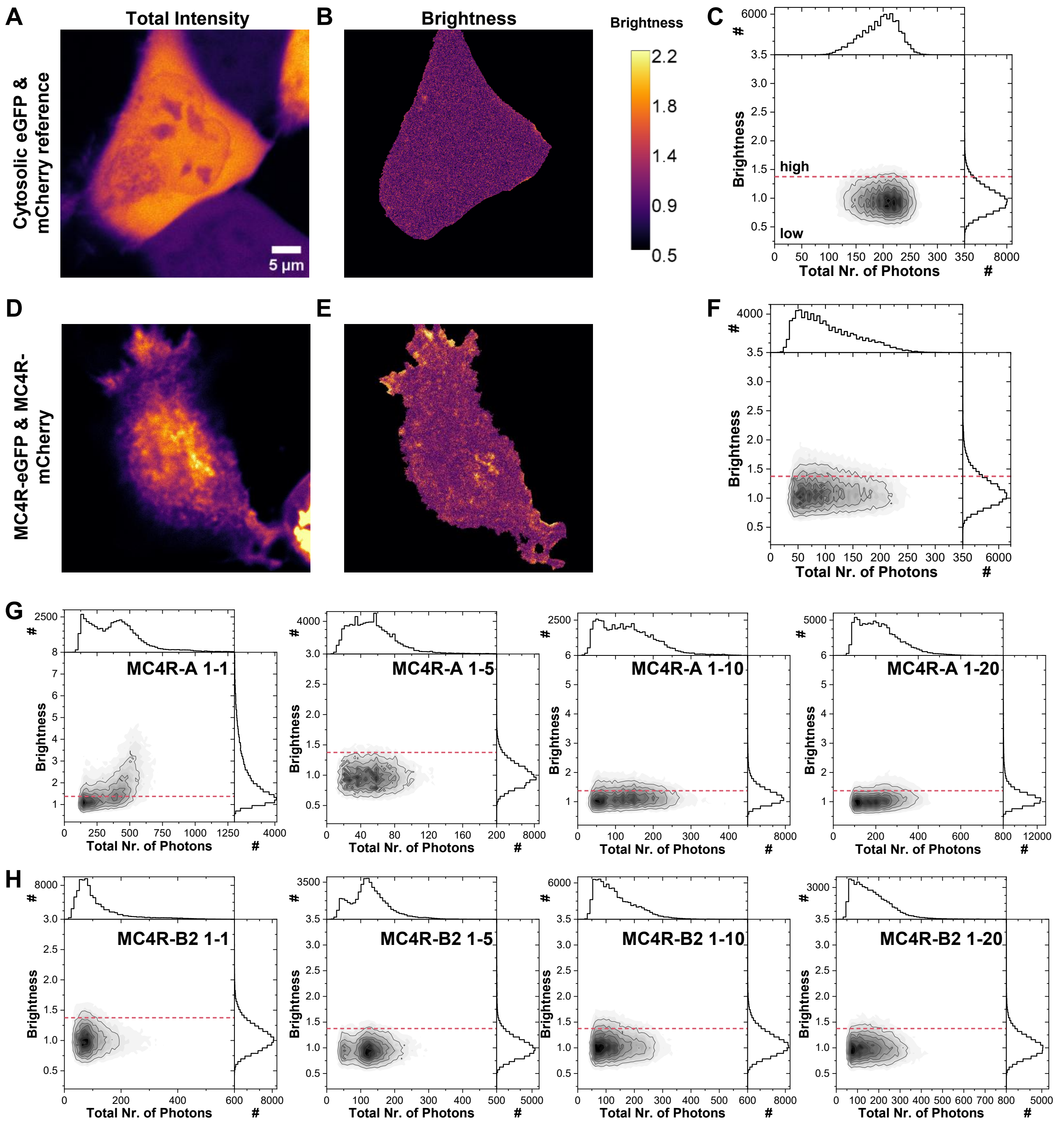

Supplementary Figure 7. Number & Brightness approach. (A) Intensity image of a cell co-transfected in a 1:1 ratio with cytosolic, monomeric eGFP and mCherry. **(B)** Brightness image of the same cell calculated based on the intensity fluctuations of all channels and time windows (green prompt, red prompt and red delay). **(C)** Pixel-wise 2D histogram of total intensity vs brightness. **(D)-(F)** Same as (A-C) for a cell co-transfected with MC4R-A-eGFP and MC4R-A-mCherry. Scale bar 5 µm. **(G)** Pixel-wise 2D histogram of total intensity vs brightness for the cells shown in **Supplementary Figure 2A**. **(H)** Pixel-wise 2D histogram of total intensity vs brightness for the cells shown in **Supplementary Figure 2B**.

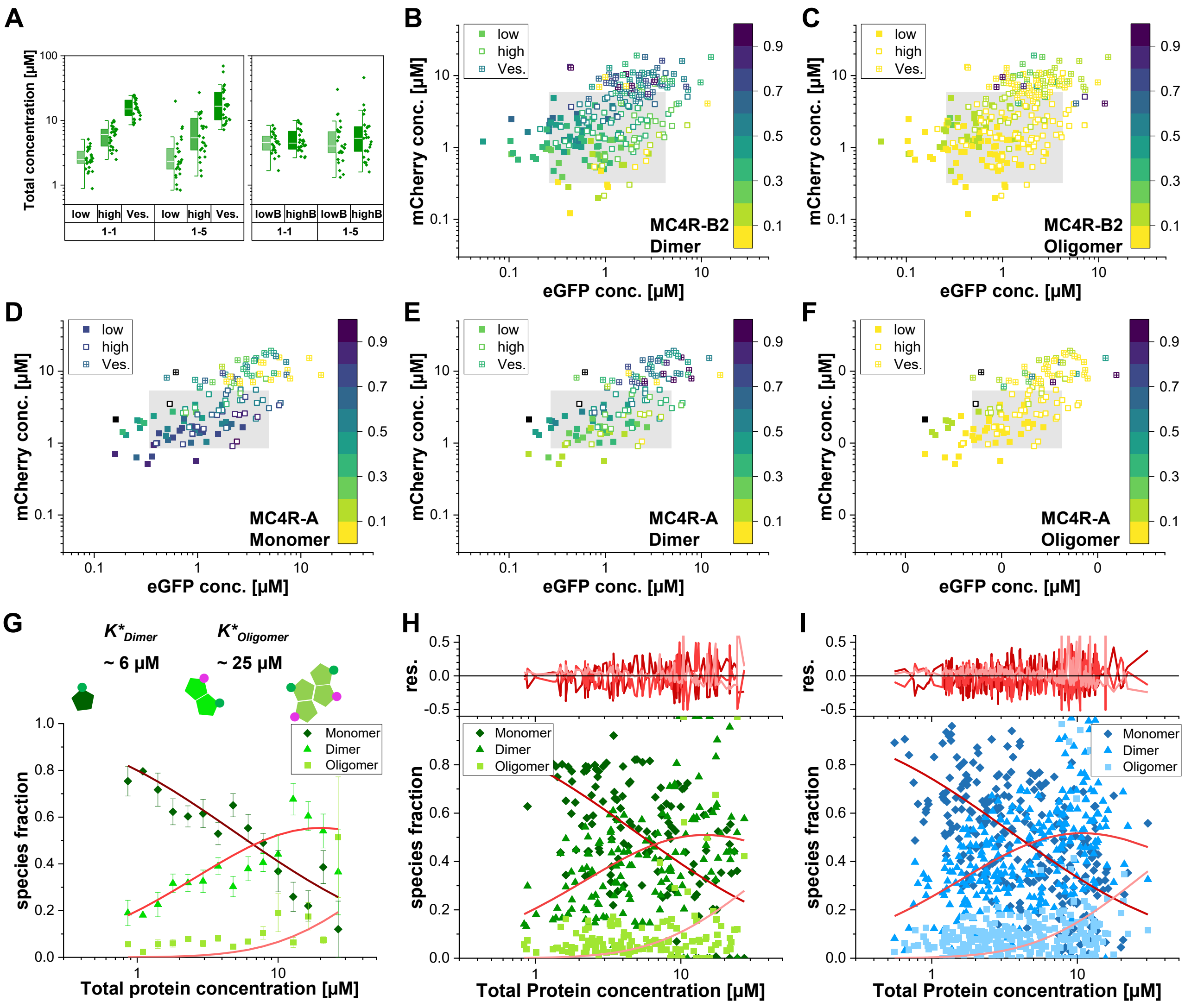

Supplementary Figure 8. Fit results for the intensity-based sub-segmentation for MC4R-A and MC4R-B2. (A) Boxplot of the total protein concentration for MC4R-A showing the accessible protein concentration distribution in the individual sub-segmented regions. **(B, C)** MC4R-B2-eGFP vs MC4R-B2-mCherry concentration color-coded by dimer (B) or oligomer (C) fraction obtained from fitting the intensity-based sub-segmentation globally with the fixed dimer and oligomer distance. Species fractions are color-coded (10 % (yellow) - 90 % (dark blue)). The shaded area shows the accessible concentration range of the 1:1 and 1:5 transfection ratio samples without the sub-segmentation. **(D) - (F)** Color-coded monomer (D), dimer (E), and oligomer (F) fractions for MC4R-A from fitting the intensity-based sub-segmentation globally with the fixed dimer and oligomer distance. **(G)** Average monomer (dark green), dimer (green), and oligomer (light green) fraction vs. total protein concentrations. Species fractions were fitted with the oligomerization model (dark red, red, and light red). Error bars show the standard error of the mean. **(H)** Species fractions of the global fit of MC4R-A were fitted with the Oligomerization model (**Eqs. S19-S21**). Color-code: Dark green/red: Monomer, green/red: dimer, light green/red: oligomer. **(I)** Same as (H) for MC4R-B2 (Dark blue/red: Monomer, blue/red: dimer, light blue/red: oligomer). The fit results for (H, I) are summarized in **Supplementary Table 6.**

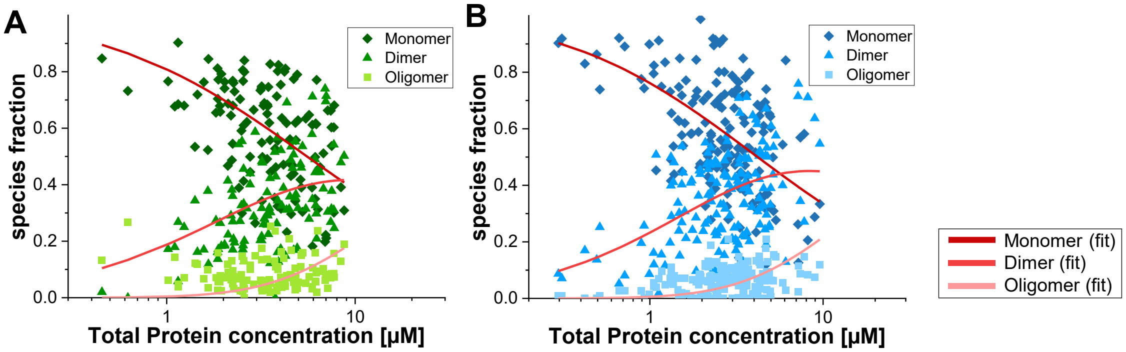

Supplementary Figure 9. Oligomerization model for MC4R-A and MC4R-B2 full cell ROIs. (A, B) Species fractions of the global two-Gaussian distances model of MC4R-A (A) and MC4R-B2 (B) of the full ROIs were fitted with the Oligomerization model (**Eqs. S19-S21**). The fit results are summarized in **Supplementary Table 6**.

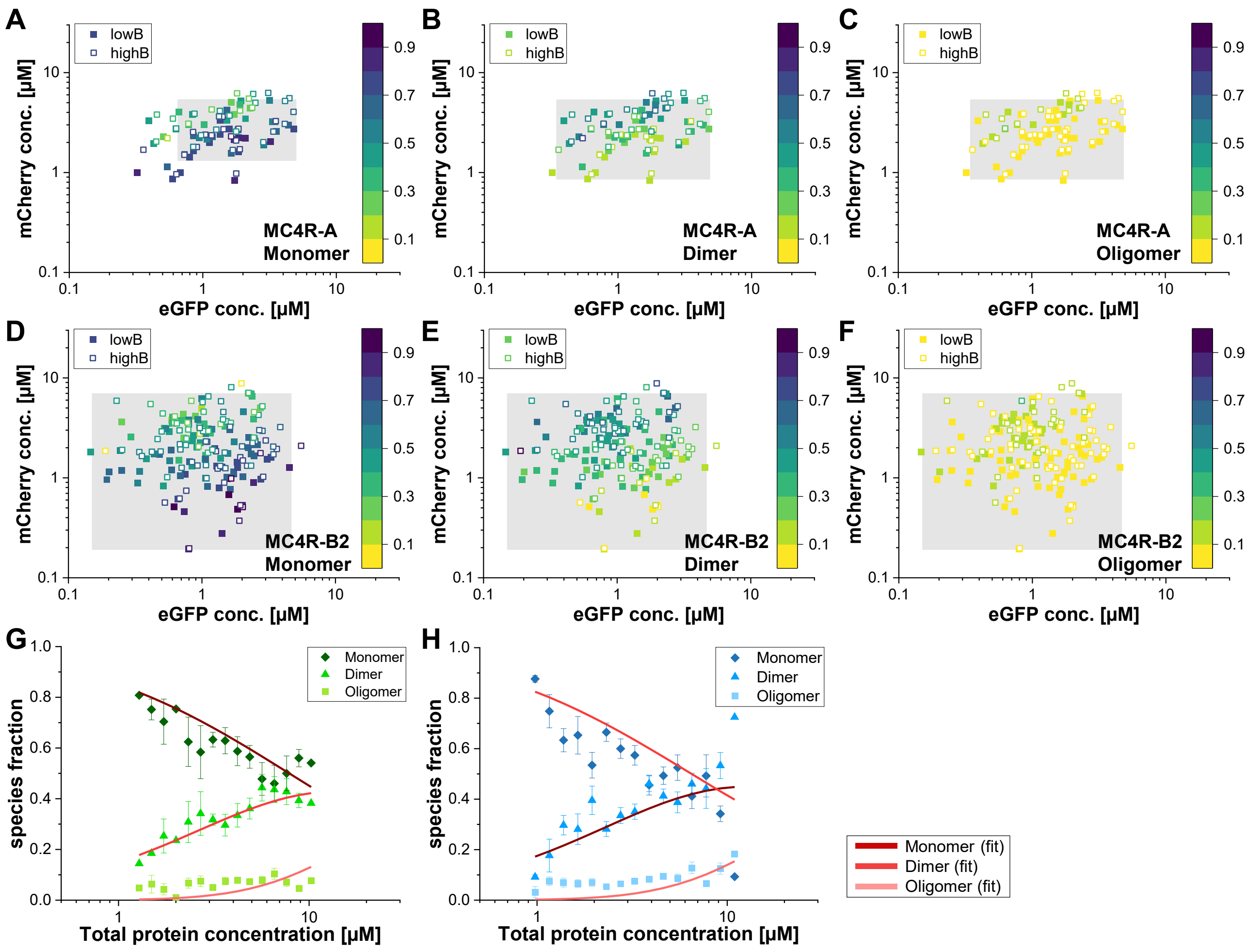

Supplementary Figure 10. Results of the Oligomerization model for the MC4R-A and MC4R-B2 brightness-based sub-segmentation. (A)-(C) MC4R-A-eGFP vs MC4R-A-mCherry concentrations color-coded for monomer (A), dimer (B) or oligomer (C) fractions obtained from fitting the brightness-based sub-segmentation globally with the fixed dimer and oligomer distance. Species fractions are color coded (10 % (yellow) - 90 % (dark blue)). The shaded area shows the accessible concentration range of the 1:1 and 1:5 transfection ratio samples without sub-segmentation. **(D)-(F)** Same as (A)-(C) for MC4R-B2. **(G)** Average monomer (dark green), dimer (green) and oligomer (light green) fractions vs the total protein concentrations obtained from the brightness-based sub-segmentation. Species fractions were fit with the Oligomerization model (**Eqs. 19-21**). Color-code: dark red, red and light red. Error bars are standard errors of the mean. **(H)** Same as (G) for MC4R-B2 (Dark blue/red: Monomer, blue/red: dimer, light blue/red: oligomer). The fit results for (G, H) are summarized in **Supplementary Table 5**. Note that $K^*_{Oligomer}$ is higher than the assessed concentration range.

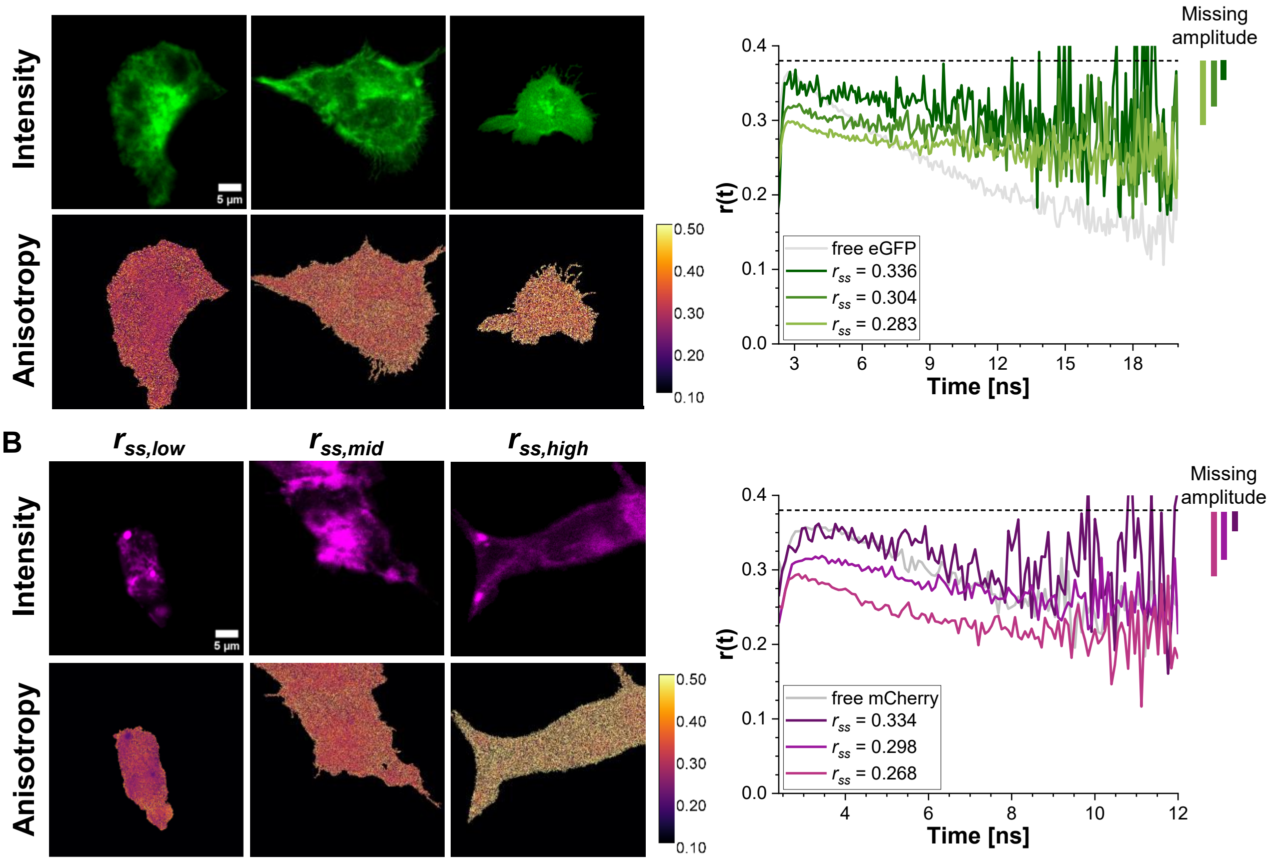

Supplementary Figure 11. Steady-state fluorescence anisotropy images of selected cells. (A) Green intensity in the "prompt" time window and steady-state anisotropy of three cells with overall low, mid and high steady-state anisotropy of cells transfected with MC4R-A-eGFP (DO). The time-resolved anisotropy is shown on the side. **(B)** Acceptor intensity in the "delay" time window and steady-state anisotropy of three cells with overall low, mid and high steady-state anisotropy of cells transfected with MC4R-B2-mCherry. The time-resolved anisotropy is shown on the side. For the green channels a g-factor of 0.946 and for the red channels a g-factor of 0.995 was used. The data were exported with a time resolution of 80 ps/bin to reduce data noise. Scale bar: 5 μm.

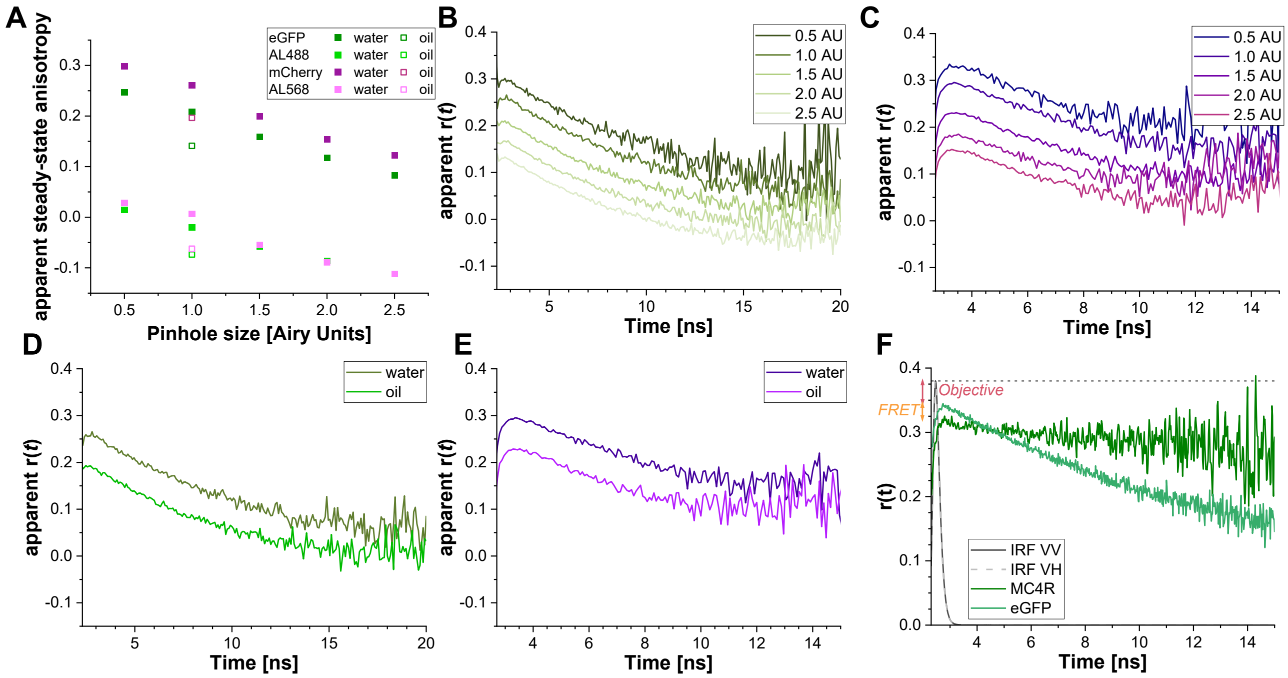

Supplementary Figure 12. Characterization of the depolarization through the microscope objective. (A) Uncorrected, apparent steady-state anisotropy of eGFP, Alexa488, mCherry and Alexa568 solutions determined for our 40x water objective (NA 1.2) or the 63x oil objective (NA1.4) and different pinhole sizes (0.5 - 2.5 Airy Units, AU). **(B)** Time-resolved anisotropies of eGFP. **(C)** Time-resolved anisotropies of mCherry. **(D)** Comparison of the apparent time-resolved anisotropy of eGFP measured with the 40x water objective (NA 1.2) and the 63x oil objective (NA 1.4). **(E)** Same as (D) for mCherry. The apparent anisotropy in panels (A) – (E) is uncorrected for the g-factor and was calculated as $r_{ss,app} = \frac{\sum I_{VV}(t) - \sum I_{VH}(t)}{\sum I_{VV}(t) + 2 * \sum I_{VH}(t)}$ and $r_{app}(t) = \frac{I_{VV}(t) - I_{VH}(t)}{I_{VV}(t) + 2 * I_{VH}(t)}$. To reduce data noise, the data was exported with a time resolution of 80 ps/bin. **(F)** For a fluorophore with a fundamental anisotropy $r_0 = 0.38$, the time-resolved anisotropy r(t) should initially be 0.38. Missing amplitudes may be due to (i) depolarization of the objective or (ii) very fast homoFRET processes. Comparing a reference dye or sample (here, an eGFP solution), in which homoFRET processes can be excluded, provides information on whether homoFRET occurs in the sample of interest (here, an MC4R measurement). For illustration, the IRF is also shown, and its height was normalized to 0.38.

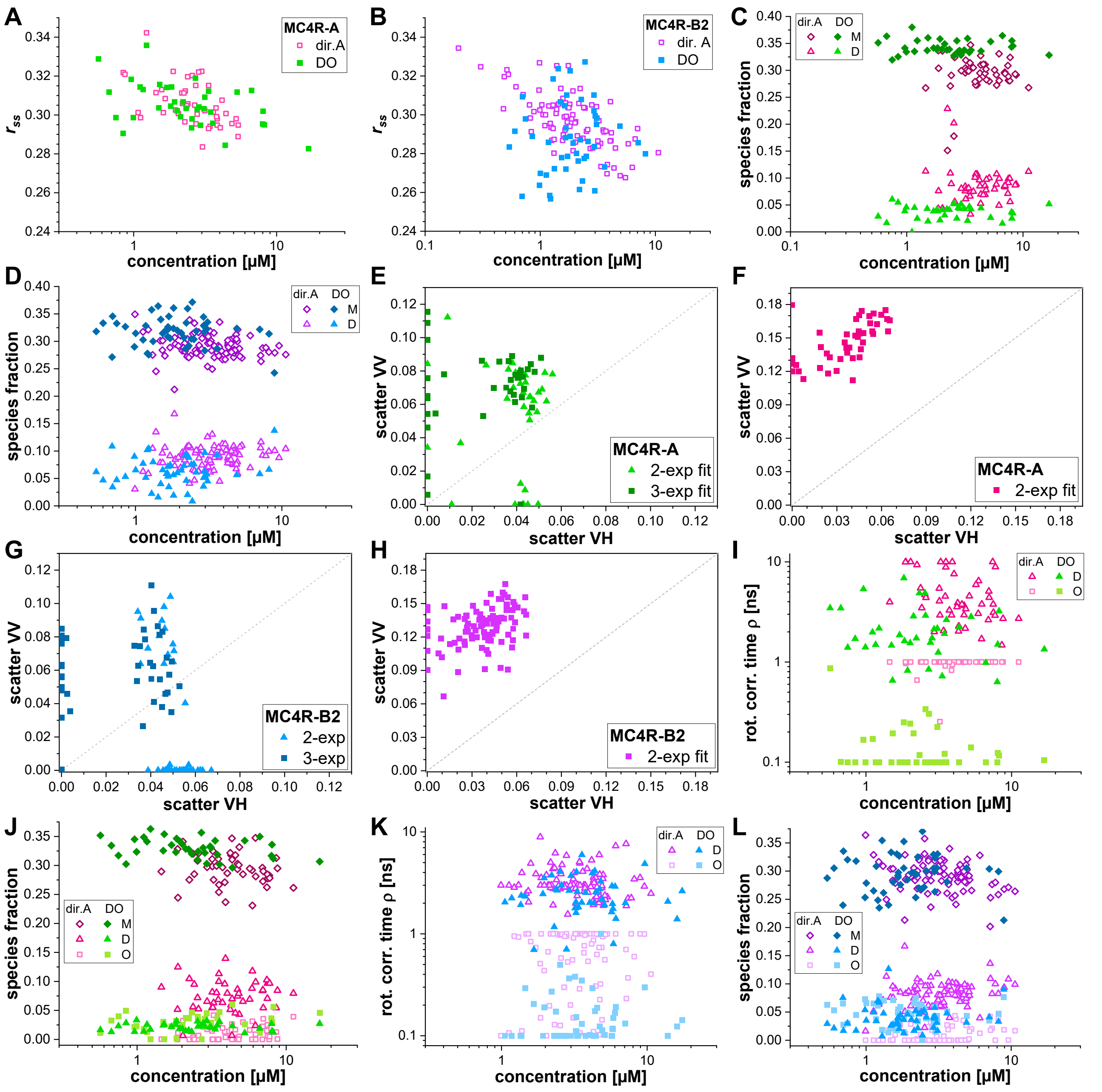

Supplementary Figure 13. Comparison of dimer and oligomer models in time-resolved anisotropy measurements. (A) Concentration-dependence of the steady-state anisotropy of DO (green) and directly excited acceptor (magenta) of the 1:1 and 1:5 co-transfected MC4R-A samples. **(B)** Same as (A) for MC4R-B2 (DO: blue, directly excited acceptor: violet). **(C)** Species fractions obtained from the dimer model for MC4R-A with a free dimer relaxation time but a fixed rotational correlation time. The concentration axis reflects the total protein concentration. **(D)** Same as (C) for MC4R-B2 (D: Dimer, M: Monomer). **(E, F)** Scatter obtained from in the dimer model (light green, DO (E); directly excited acceptor (F)) or the oligomer model (dark green, DO, (E)) in the parallel (VV) and perpendicular (VH) detection channels for MC4R-A. **(G, H)** Same as (E-F) for MC4R-B2. **(I, J)** Relaxation times (I) and species fractions (J) from the oligomer model with freely floating Oligomer and Dimer relaxation times (fixed molecule rotation) for MC4R-A (D: Dimer, M: Monomer, O: Oligomer). **(K, L)** Same as (I-J) for MC4R-B2.

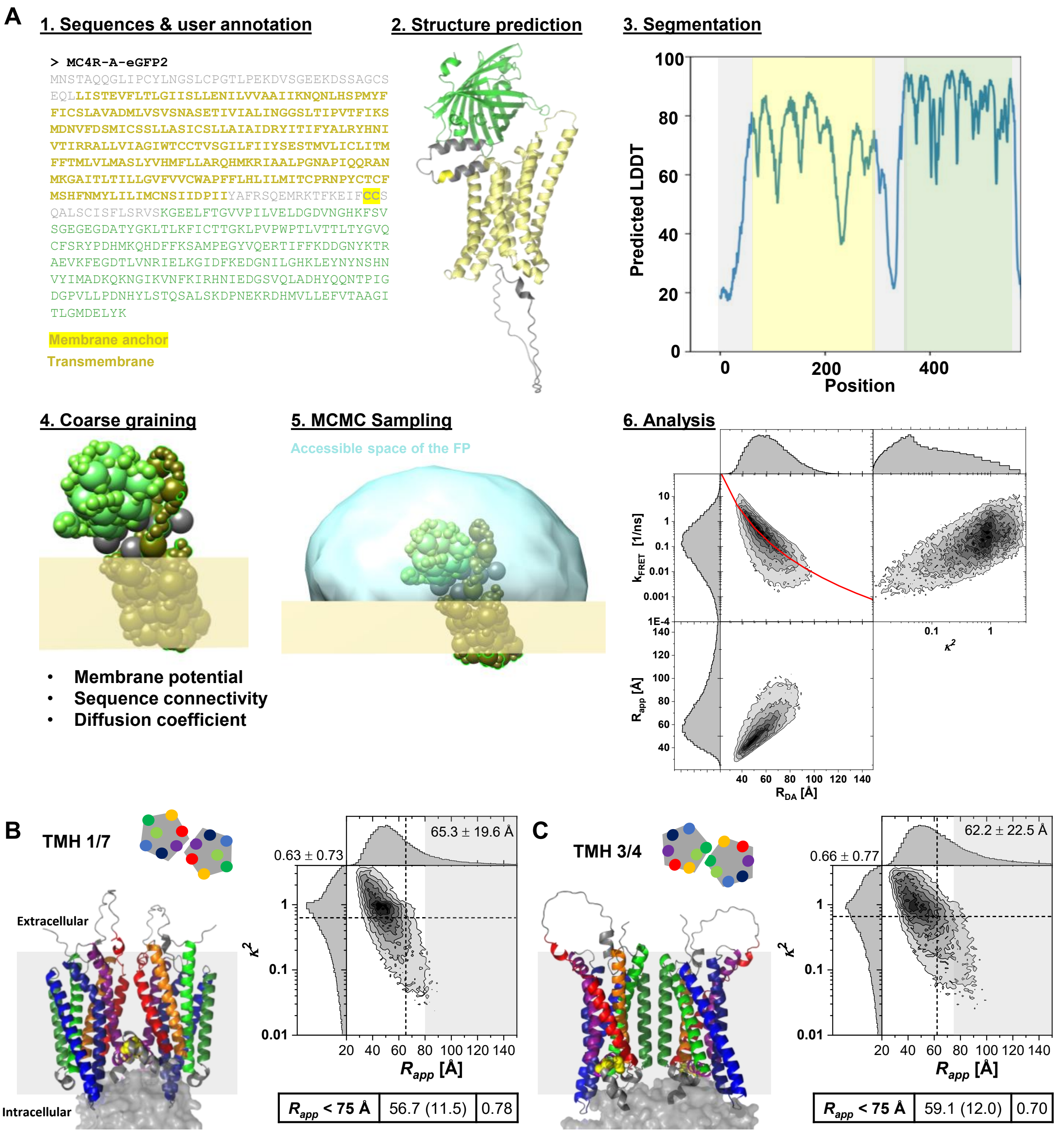

Supplementary Figure 14. Simulation workflow to estimate the spatial fluorescent proteins distribution on target proteins and resulting FRET efficiencies for protein complexes. (**A**) The fluorescent protein simulation implemented in FPSIMP (https://github.com/fluorescence-tools/fpsimp) follows a 6-step procedure: **(1)** In a first step the complete sequence of the protein of interest and the fluorescent protein is entered and annotated, e.g. transmembrane regions or membrane anchors are defined. **(2)** The structure of the tagged proteins is predicted using AlphaFold3. (3) Rigid elements (helices, β-sheets) and flexible regions are identified based on their predicted LDDT (local distance difference test), which describes the per-residue local confidence of the generated structure. **(4)** The integrative modeling platform (IMP) is used to add information such as membrane potential (i.e. such that the membrane anchor residues will stay close to/in the membrane) and to create a coarse-grained

model. **(5)** The coarse-grained model is used in Markov-Chain Monte-Carlo (MCMC) sampling to determine the accessible space of the fluorescent protein (FP, light blue halo). Here, > 10 000 structures / orientations are generated. **(6)** Using the position and the orientation of the chromophore in eGFP and mCherry, the inter-fluorophore distance distribution, R_{DA}, and the orientation factor distribution, κ^2, can be obtained. R_{app} describes the distance obtained from fitting. The red line indicates the observed distance for $\kappa^2 = 2/3$. (**B, C**) AlphaFold3 predicts different dimerization interfaces for MC4R-A. Simulated apparent inter-fluorophore distances for the TMH 1/7 (B) and the TMH 3/4 (C) dimerization model. The table indicates the distribution mean and width (in brackets) when assigning all models with R_{app} > 75 Å to x_{noFRET} (22-30%). The color code of helices is identical to **Figure 6D** in the main text. eGFP and mCherry are shown as semitransparent surface and the gray shaded area indicates the cell membrane. Dashed lines: Mean values for κ^2 and R_{app}, numbers in the 2D histogram report mean and width of the distributions.

2.2 Supplementary Tables

Supplementary Table 1. Amino acid sequences for the MC4R-A and MC4R-B2 fluorescent protein tagged constructs. The eGFP (green) and mCherry (magenta) sequences are highlighted. Orange residues mark the membrane anchor in MC4R-A, bold sequences mark the intracellular helix VIII andflexible intracellular residues.

Construct	***Sequence***
MC4R-A-eGFP	MNSTAQQGLI PCYLNGSLCP GTLPEKDVSG EEKDSSAGCS EQLLISTEVF<br>LTLGIISLLE NILVVAAIIK NQNLHSPMYF FICSLAVADM LVSVSNASET<br>IVIALINGGS LTIPVTFIKS MDNVFDSMIC SSLLASICSL LAIAIDRYIT<br>IFYALRYHNI VTIRRALLVI AGIWTCCTVS GILFIIYSES TMVLICLITM<br>FFTMLVLMAS LYVHMFLLAR QHMKRIAALP GNAPIQQRAN MKGAITLTIL<br>LGVFVVCWAP FFLHLILMIT CPRNPYCTCF MSHFNMYLIL IMCNSIIDPI<br>I**YAFRSQEMR KTFKEIF**CC**S QALSCISFLS RVS**KGEELFT GVVPILVELD<br>GDVNGHKFSV SGEGEGDATY GKLTLKFICT TGKLPVPWPT LVTTLTYGVQ<br>CFSRYPDHMK QHDFFKSAMP EGYVQERTIF FKDDGNYKTR AEVKFEGDTL<br>VNRIELKGID FKEDGNILGH KLEYNYNSHN VYIMADKQKN GIKVNFKIRH<br>NIEDGSVQLA DHYQQNTPIG DGPVLLPDNH YLSTQSALSK DPNEKRDHMV<br>LLEFVTAAGI TLGMDELYK
MC4R-A-mCherry	MNSTAQQGLI PCYLNGSLCP GTLPEKDVSG EEKDSSAGCS EQLLISTEVF<br>LTLGIISLLE NILVVAAIIK NQNLHSPMYF FICSLAVADM LVSVSNASET<br>IVIALINGGS LTIPVTFIKS MDNVFDSMIC SSLLASICSL LAIAIDRYIT<br>IFYALRYHNI VTIRRALLVI AGIWTCCTVS GILFIIYSES TMVLICLITM<br>FFTMLVLMAS LYVHMFLLAR QHMKRIAALP GNAPIQQRAN MKGAITLTIL<br>LGVFVVCWAP FFLHLILMIT CPRNPYCTCF MSHFNMYLIL IMCNSIIDPI<br>I**YAFRSQEMR KTFKEIF**CC**S QALSCISFLS RVS**KGEEDNM AIIKEFMRFK<br>VHMEGSVNGH EFEIEGEGEG RPYEGTQTAK LKVTKGGPLP FAWDILSPQF<br>MYGSKAYVKH PADIPDYLKL SFPEGFKWER VMNFEDGGVV TVTQDSSLQD<br>GEFIYKVKLR GTNFPSDGPV MQKKTMGWEA SSERMYPEDG ALKGEIKQRL<br>KLKDGGHYDA EVKTTYKAKK PVQLPGAYNV NIKLDITSHN EDYTIVEQYE<br>RAEGRHSTGG MDELYK
MC4R-B2-eGFP	MNSTAQQGLI PCYLNGSLCP GTLPEKDVSG EEKDSSAGCS EQLLISTEVF<br>LTLGIISLLE NILVVAAIIK NKNLHSPKYF FICSLAVADM LVSVSNASET<br>IVIALFNGGS LTIPVTFIKS MDNVFNSMIC SSLLASICSL LAIAIDRYIT<br>IFYALRYHDI VTIRRALLVI GSIWTCCTVS GILFIIYSES TVVLICLITM<br>SFTVLVLMAS LYVHMFLLAR QHMKRIGALP GNAAIQQRAN MKGAITLTIL<br>LGVFVFCWAP FFLHLILMIT CPRNPYCTCF MSHFNMYLIL IMCNSVIDPI<br>I**YAFRSQEMR KTFKKIFSQA RVLAFCETLQ VVHLSRVS**KG EELFTGVVPI<br>LVELDGDVNG HKFSVSGEGE GDATYGKLTL KFICTTGKLP VPWPTLVTTL<br>TYGVQCFSRY PDHMKQHDFF KSAMPEGYVQ ERTIFFKDDG NYKTRAEVKF<br>EGDTLVNRIE LKGIDFKEDG NILGHKLEYN YNSHNVYIMA DKQKNGIKVN<br>FKIRHNIEDG SVQLADHYQQ NTPIGDGPVL LPDNHYLSTQ SALSKDPNEK<br>RDHMVLLEFV TAAGITLGMD ELYK
MC4R-B2-mCherry	MNSTAQQGLI PCYLNGSLCP GTLPEKDVSG EEKDSSAGCS EQLLISTEVF<br>LTLGIISLLE NILVVAAIIK NKNLHSPKYF FICSLAVADM LVSVSNASET<br>IVIALFNGGS LTIPVTFIKS MDNVFNSMIC SSLLASICSL LAIAIDRYIT<br>IFYALRYHDI VTIRRALLVI GSIWTCCTVS GILFIIYSES TVVLICLITM<br>SFTVLVLMAS LYVHMFLLAR QHMKRIGALP GNAAIQQRAN MKGAITLTIL<br>LGVFVFCWAP FFLHLILMIT CPRNPYCTCF MSHFNMYLIL IMCNSVIDPI<br>I**YAFRSQEMR KTFKKIFSQA RVLAFCETLQ VVHLSRVS**KG EEDNMAIIKE<br>FMRFKVHMEG SVNGHEFEIE GEGEGRPYEG TQTAKLKVTK GGPLPFAWDI<br>LSPQFMYGSK AYVKHPADIP DYLKLSFPEG FKWERVMNFE DGGVVTVTQD<br>SSLQDGEFIY KVKLRGTNFP SDGPVMQKKT MGWEASSERM YPEDGALKGE<br>IKQRLKLKDG GHYDAEVKTT YKAKKPVQLP GAYNVNIKLD ITSHNEDYTI<br>VEQYERAEGR HSTGGMDELY K

Supplementary Table 2. Summary of biological and technical replicates. Number of collected cell images and number of used cells (X/Y) is shown per experiment – each entry represents a separate transfection experiment performed on a separate day.

Construct	*DO*	*1-1*	*1-5*	*1-10*	*1-20*
**MC4R-A**	7/7, 7/7, 8/8, 8/8	6/6, 8/8, 7/6, 7/6, 3/3, 3/3, 4/4, 4/4	7/7, 7/7, 6/5	8/7, 8/8, 8/8, 8/8	7/5, 8/7, 8/8, 8/6
**MC4R-B2**	8/8, 8/8, 8/8, 9/9, 7/7, 8/8	6/5, 6/6, 8/8, 6/6, 10/10, 8/7, 4/4, 3/3, 4/4, 4/4	10/10, 8/7, 8/8, 6/6, 8/8, 9/9	8/6, 8/8, 8/8, 8/8	7/5, 8/7, 8/8, 8/8

Supplementary Table 3. Overview of fit models used in the heteroFRET analysis. In the heteroFRET analysis the eGFP signal from either singly transfected (DO control) or co-transfected HEK293T cells (DA) was used.

Construct	*DO*	*DA*
**Multiexponential Lifetimes**	eq. S1/2, m=2	eq. S1/2, m=3
**Single Gaussian Distance (ROIs)**	n.a.	eq. S3-S5<br>$\bar{R}_{app}$, $\sigma_{app} = 5 - 25$ Å
**Two Gaussian Distances – free (ROIs)**	n.a.	eq. S3-S5<br>$\bar{R}_{di}$, $\sigma_{di} = 6$ Å, $\bar{R}_{ol}$, $\sigma_{ol} = 1$ Å
**Two Gaussian Distances – global (ROIs & sub-ROIs)**	n.a.	eq. S3-S5, $\sigma_{di} = 5 - 25$ Å, $\sigma_{ol} = 1$ Å<br>MC4R-A: $\bar{R}_{di} = 60.4$ Å, $\bar{R}_{ol} = 37.5$ Å<br>MC4R-B2: $\bar{R}_{di} = 58.7$ Å, $\bar{R}_{ol} = 37.4$ Å

Supplementary Table 4. Overview of fit models used in the homoFRET analysis. In the homoFRET analysis the eGFP signal from singly transfected (DO control) HEK293T cells or directly excited acceptor signal of co-transfected HEK293T cells (dir.A) was used.

Construct	*DO*	*Dir.A*
**Diner model – free (eq. S7)**	$\rho_{global} = 100$ ns, ρ_{di}	$\rho_{global} = 100$ ns
**Dimer model – global (eq. S7)**	$\rho_{global} = 100$ ns,<br>MC4R-A: $\rho_{di} = 0.74\ ns$<br>MC4R-B2: $\rho_{di} = 0.72\ ns$	$\rho_{global} = 100$ ns<br>MC4R-A: $\rho_{di} = 4.32\ ns$<br>MC4R-B2: $\rho_{di} = 3.33\ ns$
**Oligomer model – free (eq. S8)**	$\rho_{global} = 100$ ns, ρ_{di}, ρ_{ol}	$\rho_{global} = 100$ ns, ρ_{di}, ρ_{ol}
**Oligomer model – global (eq. S8)**	$\rho_{global} = 100$ ns,<br>MC4R-A: $\rho_{di} = 3.09\ ns$,<br>$\rho_{ol} = 0.22\ ns$<br>MC4R-B2: $\rho_{di} = 2.58\ ns$,<br>$\rho_{ol} = 0.19\ ns$	Analysis was not performed.

Supplementary Table 5. The binned species fractions from the global two-Gaussian distance model for full ROIs and the intensity- and brightness-based sub-segmentation of MC4R-A and MC4R-B2 were fitted with the Oligomerization model. 95% confidence intervals (CI) were determined by 500 rounds of bootstrapping. [] indicates CI.

Parameter	***MC4R-A (full ROI)***	***MC4R-A Intensity***	***MC4R-A Brightness***	***MC4R-B2 (full ROI)***	***MC4R-B2 Intensity***	***MC4R-B2 Brightness***
Used data	1-1, 1-5, 1-10, 1-20	1-1, 1-5	1-1, 1-5	1-1, 1-5, 1-10, 1-20	1-1, 1-5	1-1, 1-5
m_{points}	39	45	45	39	45	45
n_{cells}	118	45	45	165	92	92
K^*_{Dimer} [μM]	5.41 [5.76-7.17]	6.29 [5.01-7.78]	8.25 [7.10 – 9.66]	4.98 [4.24 – 5.79]	4.91 [3.76 – 7.20]	5.04 [3.82 – 6.27]
$K^*_{Oligomer}$ [μM]	13.2 [6.25 – 20.4]	24.5 [8.72 – 60.0]	14.6 [8.23 – 19.5]	17.5 [6.83 – 28.0]	27.6 [15.3 – 35.0]	15.3 [8.20 – 21.9]

Supplementary Table 6. The species fractions from the global two-Gaussian distance model for full ROIs and the intensity- and brightness-based sub-segmentation of MC4R-A and MC4R-B2 were fitted with the Oligomerization model. 95% confidence intervals (CI) determined by 500 rounds of bootstrapping. [] indicates CI.

Parameter	***MC4R-A (full ROI)***	***MC4R-A Intensity***	***MC4R-A Brightness***	***MC4R-B2 (full ROI)***	***MC4R-B2 Intensity***	***MC4R-B2 Brightness***
Used data	1-1, 1-5, 1-10, 1-20	1-1, 1-5	1-1, 1-5	1-1, 1-5, 1-10, 1-20	1-1, 1-5	1-1, 1-5
m_{points}	354	402	270	495	810	552
K^*_{Dimer} [μM]	6.94 [5.86 – 8.26]	6.18 [5.05 – 7.44]	7.93 [6.37 – 9.75]	5.01 [4.29 – 5.87]	4.36 [3.76 – 5.02]	5.33 [4.56 – 6.20]
$K^*_{Oligomer}$ [μM]	8.77 [6.94 – 10.7]	25.0 [13.1 – 44.5]	12.1 [9.79 – 14.5]	9.37 [7.25 – 12.3]	18.1 [13.1 – 24.2]	10.1 [8.49 – 11.9]

Supplementary Table 7. The species fractions obtained from the time-resolved anisotropy analysis of MC4R-A and MC4R-B2 were fitted with the Dimerization model. CI = 95% confidence interval as determined by 500 rounds of bootstrapping. [] indicates CI.

Parameter	***MC4R-A (Anisotropy)***	***MC4R-B2 (Anisotropy)***
Used data	1-1, 1-5, DO	1-1, 1-5, DO
n_{cells}	85	140
K^*_{Dimer} [μM]	26.4 [22.8 – 30.4]	15.3 [13.7 – 17.0]

Supplementary References

Balakrishnan A, Hemmen K, Choudhury S, Krohn JH, Jansen K, Friedrich M, et al. 2022. Unraveling the hidden temporal range of fast beta(2)-adrenergic receptor mobility by time-resolved fluorescence. Commun Biol. Feb 28;5:176. Epub 2022/03/02.

Berg S, Kutra D, Kroeger T, Straehle CN, Kausler BX, Haubold C, et al. 2019. ilastik: interactive machine learning for (bio)image analysis. Nat Methods. Dec;16:1226-1232. Epub 2019/10/02.

Digman MA, Dalal R, Horwitz AF, Gratton E. 2008. Mapping the number of molecules and brightness in the laser scanning microscope. Biophys J. Mar 15;94:2320-2332. Epub 2007/12/22.

Erdelyi M, Simon J, Barnard EA, Kaminski CF. 2014. Analyzing Receptor Assemblies in the Cell Membrane Using Fluorescence Anisotropy Imaging with TIRF Microscopy. Plos One. Jun 19;9.

Greife A, Felekyan S, Ma QJ, Gertzen CGW, Spomer L, Dimura M, et al. 2016. Structural assemblies of the di- and oligomeric G-protein coupled receptor TGR5 in live cells: an MFIS-FRET and integrative modelling study. Sci Rep-Uk. Nov 11;6.

Hemmen K, Choudhury S, Friedrich M, Balkenhol J, Knote F, Lohse MJ, et al. 2021. Dual-Color Fluorescence Cross-Correlation Spectroscopy to Study Protein-Protein Interaction and Protein Dynamics in Live Cells. J Vis Exp. Dec 11. Epub 2021/12/28.

Koshioka M, Sasaki K, Masuhara H. 1995. Time-Dependent Fluorescence Depolarization Analysis in 3-Dimensional Microspectroscopy. Appl Spectrosc. Feb;49:224-228.

Kravets E, Degrandi D, Ma Q, Peulen TO, Klumpers V, Felekyan S, et al. 2016. Guanylate binding proteins directly attack Toxoplasma gondii via supramolecular complexes. Elife. Jan 27;5. Epub 2016/01/28.

Lambert TJ. 2019. FPbase: a community-editable fluorescent protein database. Nat Methods. Apr;16:277-278. Epub 2019/03/20.

Li CH, Lee CK. 1993. Minimum Cross Entropy Thresholding. Pattern Recogn. Apr;26:617-625.

Li CH, Tam PKS. 1998. An iterative algorithm for minimum cross entropy thresholding. Pattern Recogn Lett. Jun;19:771-776.

Otsu N. 1979. Threshold Selection Method from Gray-Level Histograms. Ieee T Syst Man Cyb.9:62-66.

Peulen TO. 2025. Exploring Time-Resolved Fluorescence Data: A Software Solution for Model Generation and Analysis. Spectroscopy Journal.3:16. Epub 1 May 2025.

Peulen TO, Hemmen K, Greife A, Webb BM, Felekyan S, Sali A, et al. 2025. tttrlib: modular software for integrating fluorescence spectroscopy, imaging, and molecular modeling. Bioinformatics. Feb 4;41. Epub 2025/01/22.

Peulen TO, Opanasyuk O, Seidel CAM. 2017. Combining Graphical and Analytical Methods with Molecular Simulations To Analyze Time-Resolved FRET Measurements of Labeled Macromolecules Accurately. J Phys Chem B. Sep 7;121:8211-8241.

Schindelin J, Arganda-Carreras I, Frise E, Kaynig V, Longair M, Pietzsch T, et al. 2012. Fiji: an open-source platform for biological-image analysis. Nature Methods. Jul;9:676-682.

Striker G, Subramaniam V, Seidel CAM, Volkmer A. 1999. Photochromicity and fluorescence lifetimes of green fluorescent protein. J Phys Chem B. Oct 7;103:8612-8617.

Thompson NL. 2002. Fluorescence Correlation Spectroscopy. In: Topics in Fluorescence Spectroscopy. Boston, MA: Springer. p. 337-378.

van der Walt S, Schonberger JL, Nunez-Iglesias J, Boulogne F, Warner JD, Yager N, et al. 2014. scikit-image: image processing in Python. PeerJ.2:e453. Epub 2014/07/16.

Widengren J, Mets U, Rigler R. 1995. Fluorescence Correlation Spectroscopy of Triplet-States in Solution - a Theoretical and Experimental-Study. J Phys Chem-Us. Sep 7;99:13368-13379.
